# Pediatric long COVID is characterized by myeloid CCR6 suppression and immune dysregulation

**DOI:** 10.1172/jci.insight.201111

**Published:** 2026-02-19

**Authors:** Jon Izquierdo-Pujol, Núria Pedreño-López, Tetyana Pidkova, Maria Nevot, Victor Urrea, Fernando Laguía, Francisco Muñoz-López, Judith Dalmau, Alba Gonzalez-Aumatell, Clara Carreras-Abad, Maria Mendez, Carlos Rodrigo, Marta Massanella, Julià Blanco, Jorge Carrillo, Benjamin Trinité, Javier Martinez-Picado, Sara Morón-López

**Affiliations:** 1 IrsiCaixa, Badalona, Barcelona, Spain.; 2 Germans Trias i Pujol Research Institute (IGTP), Badalona, Spain.; 3 Biomedicine doctoral programme, Faculty of Biology, University of Barcelona, Barcelona, Spain.; 4 Department of Pediatrics, Germans Trias i Pujol University Hospital, Badalona, Spain.; 5 Department of Pediatrics, Obstetrics and Gynecology, Preventive Medicine and Public Health, Faculty of Medicine, Universitat Autònoma de Barcelona, Cerdanyola del Vallès, Spain.; 6 CIBERINFEC, Madrid, Spain.; 7 Department of Infectious Disease and Immunity, University of Vic-Central University of Catalonia (UVic-UCC), Vic, Spain.; 8 Catalan Institution for Research and Advanced Studies (ICREA), Barcelona, Spain.

**Keywords:** Clinical Research, Immunology, Infectious disease, Adaptive immunity, COVID-19, Innate immunity

## Abstract

The biological mechanisms underlying long COVID in the pediatric population are poorly understood. Our study aimed to characterize the immune pathophysiology of long COVID in this population. We analyzed major immune cell compartments in PBMCs and the specific SARS-CoV-2 antibody response in 99 patients with long COVID and in 18 patients without long COVID at 3 months after acute infection. Our findings indicate that pediatric long COVID is associated with a dysregulated immune response characterized by altered innate immunity and overactivated T, B, and NK cell responses. Furthermore, young people with long COVID had an impaired humoral response to SARS-CoV-2 marked by a dysregulated B cell compartment and lower levels of anti-RBD IgG and IgA. This correlated with reduced neutralizing capacity against SARS-CoV-2. Random forest analysis identified CCR6 expression on myeloid cells as the most relevant biomarker that distinguishes individuals with long COVID from control individuals with 79% accuracy.

## Abstract 2

We examined blood immune cells from kids with and without long COVID. Overactive immunity and weak COVID antibodies link to long COVID. CCR6 on myeloid cells correctly classifies it with 79% accuracy.

## Graphical abstract

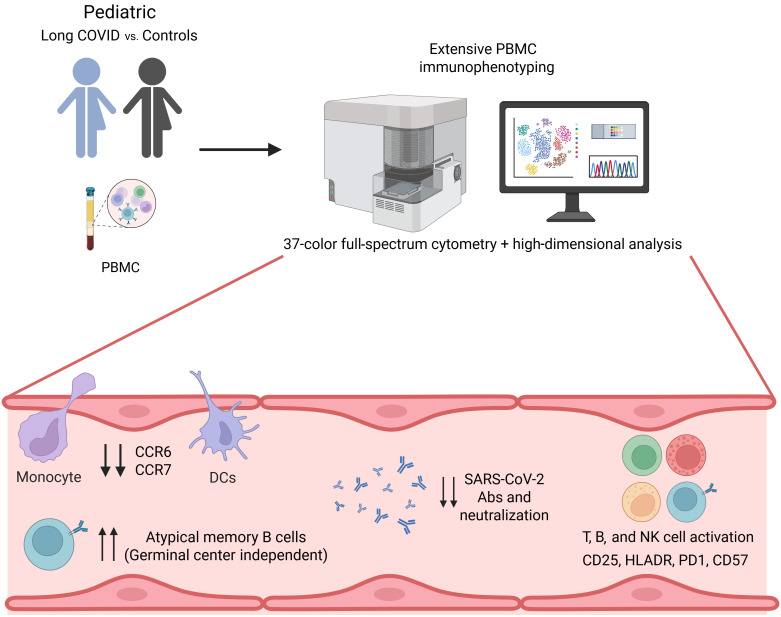

## Introduction

The emergence of SARS-CoV-2, the etiological agent of COVID-19, led to the declaration of a global pandemic in March 2020. Although most individuals infected with SARS-CoV-2 recover, a substantial proportion (approximately 10%–20%) experience persistent symptoms lasting beyond the acute phase of infection, a condition referred to as long COVID (LC) or post-COVID-19 condition (1, 2). According to the WHO, LC is defined as a condition occurring in individuals with a history of probable or confirmed SARS CoV-2 infection, typically within 3 months of the onset of COVID-19, with symptoms persisting for at least 2 months and not explained by an alternative diagnosis (2). Although methodological variation among studies has led to inconsistent prevalence estimates across age groups, available evidence indicates that approximately 65 million people worldwide have already experienced LC (3), a burden expected to grow in the coming years. LC affects not only adults but has also been extensively reported in children and young people (4–7).

Recent research on LC has focused primarily on unraveling its underlying pathophysiological mechanisms. Studies have demonstrated associations with immune dysregulation (8, 9), autoimmunity (10, 11), SARS-CoV-2 antigen persistence (12, 13), and viral reactivation (10, 14). However, knowledge of this condition in the pediatric population remains limited, as most LC studies focus on the adult population, with minimal attention given to understanding the potential driving mechanisms in pediatric patients (15).

The immune system of children and young people differs from that of adults in terms of maturation, regulation, and antiviral responses, influencing how they react to viral infections such as SARS-CoV-2 (16). Determining whether LC in children and young people shares immunological features with LC in adults or represents a distinct entity is essential for developing targeted strategies for monitoring and treatment. We therefore hypothesized that children and young people with LC would display immune signatures indicative of sustained immune activation and altered B and T cell memory, potentially associated with markers of viral antigen persistence. To test this hypothesis, we conducted comprehensive immunophenotyping of PBMCs using a 37-color full-spectrum cytometry panel, quantified anti–SARS-CoV-2 antibody levels and neutralization capacity, and assessed the persistence of SARS-CoV-2 antigens and RNA in plasma from pediatric participants with and without LC.

## Results

### Characteristics of the pediaCOVID cohort.

The pediaCOVID cohort (5, 17, 18) is composed of 2 groups: the LC group, comprising 99 patients diagnosed with LC, and a control group, comprising 18 individuals without LC or any other inflammatory disease or acute infection. The clinical characteristics of both groups are described in Table 1. Fatigue was the most common symptom in the LC group (98.0%), followed by headache (75.8%) and dyspnea (63.6%). Neurological symptoms, including headache, brain fog, poor concentration, and difficulties with attention and planning, were especially frequent. Moreover, patients with LC had a median of 12 persistent symptoms. Overall, the clinical presentation in children and young people resembled that seen in adults.

### Deep immunophenotyping of PBMCs reveals immune dysregulation in children and young people with LC.

To investigate how peripheral immune dysregulation is linked to LC, a 37-color full-spectrum cytometry panel was used to comprehensively profile peripheral immune cell populations in young patients with and without LC. Major immune cell subsets — myeloid cells (including DCs and monocytes), NK cells, T cells, and B cells — were identified by manual gating using lineage markers (Supplemental Figure 1; supplemental material available online with this article; https://doi.org/10.1172/jci.insight.201111DS1). Each subset was then analyzed with FlowSOM to characterize and annotate clusters. In total, 80 million cells were analyzed: 15% myeloid cells, 12% NK cells, 65% T cells, and 8% B cells (Supplemental Table 6).

### Myeloid cells are dysregulated in young patients with LC.

**Figure 1:**
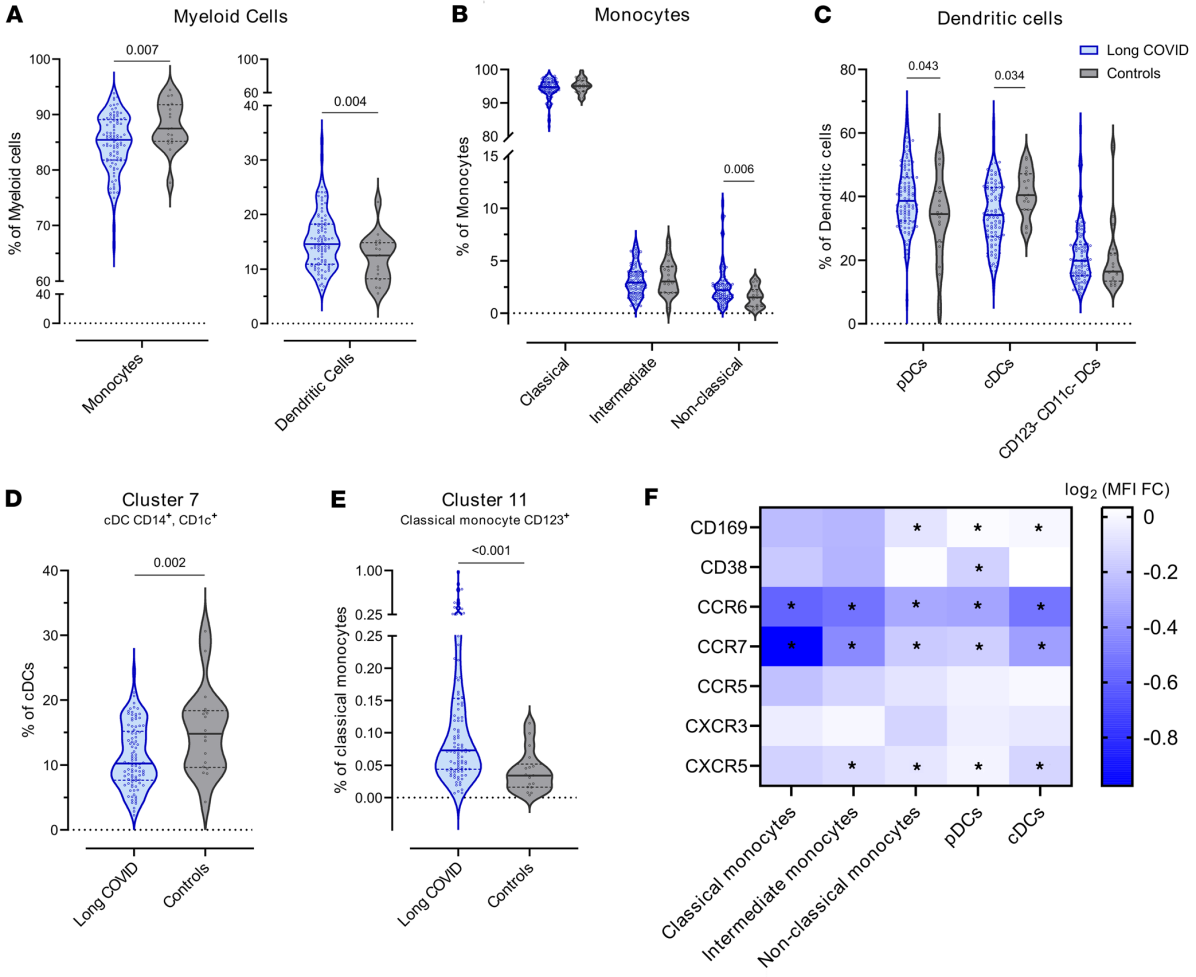
Myeloid cell compartment in children and young people with and without LC. (**A**) Frequency of monocytes (*P* = 0.007, FDR = 0.007) and DCs (*P* = 0.004, FDR = 0.007) with respect to total myeloid cells among the LC and control cohorts. (**B**) Frequency of classical (*P* nonsignificant), intermediate (*P* nonsignificant), and nonclassical (*P* = 0.006, FDR = 0.030) monocytes with respect to total monocytes among the LC and control cohorts. (**C**) Frequency of pDCs (*P* = 0.043, FDR = 0.071), cDCs (*P* = 0.034, FDR = 0.071), and CD123^–^ CD11c^–^ DCs (*P* nonsignificant) with respect to total DCs in the LC and control cohorts. (**D**) Frequency of cluster 7 with respect to total cDCs among the LC and control cohorts (*P* = 0.002, FDR = 0.014). (**E**) Frequency of cluster 11 with respect to total classical monocytes in the LC and control cohorts (*P*< 0.001, FDR = 0.001). (**F**) MFI fold-change (FC) of CD169, CD38, CCR6, CCR7, CCR5, CXCR3, and CXCR5 in the LC and control cohorts in each of the myeloid cell populations. *P < 0.05, FDR < 0.05. The frequency of each cluster and the MFI of the different markers was compared between the LC and control cohorts using linear regression models adjusted for SARS-CoV-2 antigen exposure, sex, and age as covariates. In all analyses, LC group, *n* = 98; control group, *n* = 18. Each dot represents an individual, and median and IQR values are indicated. *P* values less than 0.05 were considered statistically significant and adjusted for multiple testing using FDR.

We identified 17 clusters in the myeloid cell population using the following markers: CD1c, CD11b, CD16, CD11c, HLADR, CD95, CD14, and CD123 (Supplemental Figure 2A, Supplemental Figure 3A, and Supplemental Table 2). Each cluster was annotated as a monocyte or DC clusters depending on the expression of various markers (Supplemental Table 6). Monocyte clusters were classified as classical, intermediate, or nonclassical, and DC clusters were classified as conventional (cDCs), plasmacytoid (pDCs), or CD11c^–^ CD123^–^ DCs (Figure 1A, Supplemental Figure 2A, and Supplemental Table 6).

Pediatric LC was associated with decreased frequency of monocytes and increased frequency of DCs (Figure 1A). Subanalysis of the 3 monocyte populations revealed increased frequency of nonclassical monocytes in the LC group (Figure 1B). The DC compartment was also dysregulated in pediatric LC, with higher proportions of pDCs and decreased cDCs (Figure 1C). Among the clusters analyzed, cluster 7 (CD14^+^, CD11c^+^, CD1c^+^) was decreased in pediatric LC, and cluster 11 (CD14^+^, CD16^–^, CD123^+^) was increased (Figure 1, D and E).

Finally, we analyzed the differential expression of various markers (CD169, CD38, CCR7, CCR6, CCR5, CXCR3, and CXCR5) between patients with and without LC (Figure 1F). Pediatric LC was associated with decreased expression of both CCR7 and CCR6 in DCs and monocyte populations. Decreased expression of CXCR5, CD169, and CD38 was associated with pediatric LC, albeit only in some DCs and monocyte populations.

To summarize, pediatric LC was associated with a dysregulated myeloid cell compartment marked by a lower frequency of monocytes that were enriched in the nonclassical subset and an increase in DCs with an overrepresentation of pDCs. Both monocytes and DCs showed lower expression of CCR6 and CCR7 than the control group.

### Pediatric LC is associated with increased NK cell activation.

**Figure 2:**
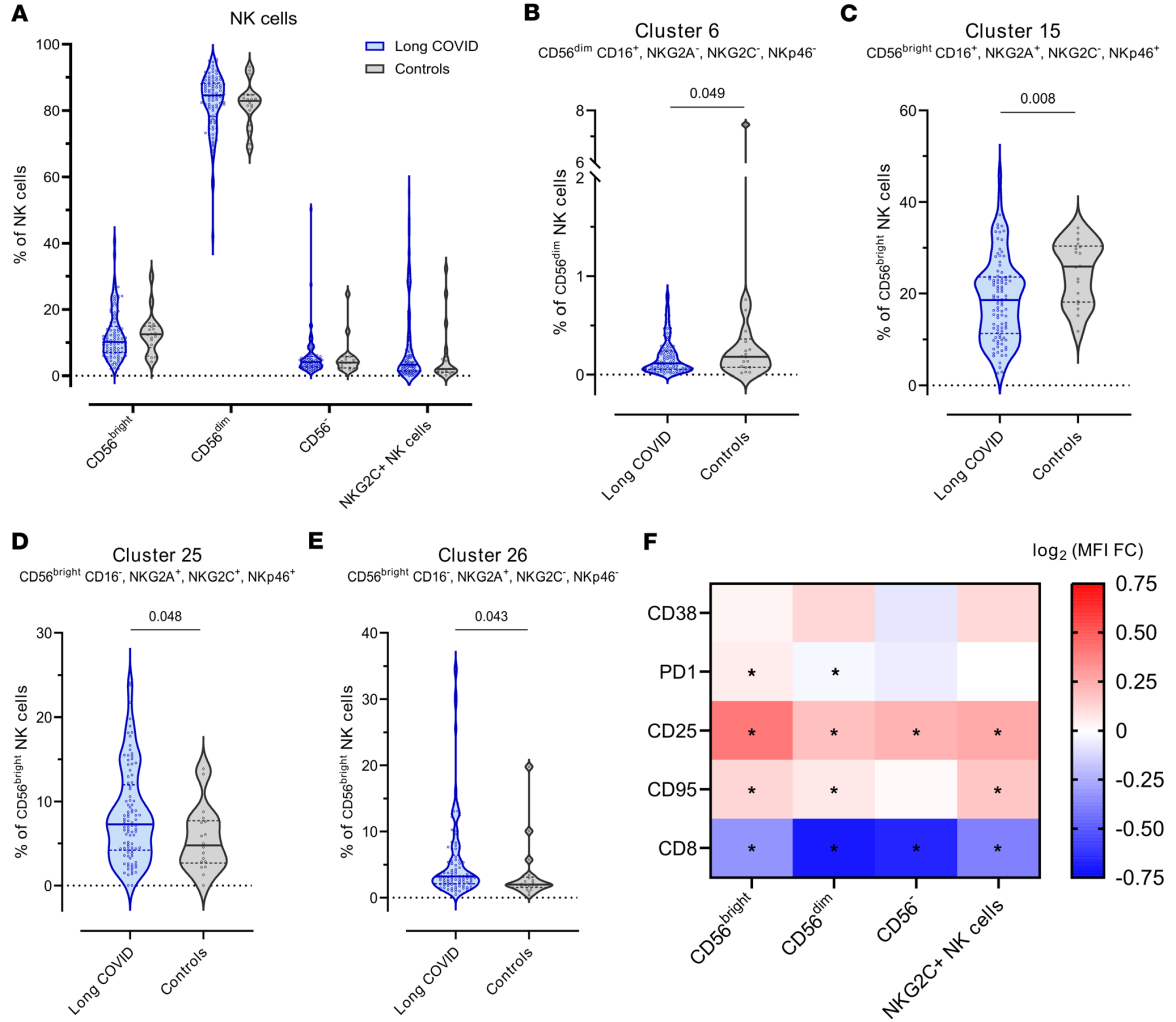
NK cell compartment in children and young people with and without LC. (**A**) Frequency of CD56^bright^, CD56^dim^, CD56^–^, and NKG2C^+^ NK cells with respect to total NK cells (all *P* nonsignificant). (**B**) Frequency of cluster 6 with respect to total CD56^dim^ NK cells in the LC and control cohorts (*P* = 0.049, FDR = 0.427). (**C**) Frequency of cluster 15 with respect to total CD56^bright^ NK cells in the LC and control cohorts (*P* = 0.008, FDR = 0.306). (**D**) Frequency of cluster 25 with respect to total CD56^bright^ NK cells in the LC and control cohorts (*P* = 0.048, FDR = 0.427). (**E**) Frequency of cluster 26 with respect to total CD56^bright^ NK cells in the LC and control cohorts (*P* = 0.043, FDR = 0.427). (**F**) MFI fold-change (FC) of CD38, PD1, CD25, CD95, and CD8 between the LC and control cohorts in each of the NK cell populations. *P < 0.05, FDR < 0.05. The frequency of each cluster and the MFI of the different markers was compared between the LC and control cohorts using linear regression models adjusted for SARS-CoV-2 antigen exposure, sex, and age as covariates. In all analyses, LC group, *n* = 98; control group, *n* = 18. Each dot represents an individual, and median and IQR values are indicated. *P* values less than 0.05 were considered statistically significant and adjusted for multiple testing using FDR.

We identified 33 clusters in the NK cell population using the following markers: CD56, CD16, CD57, NKG2A, NKG2C, NKp46, CCR5, and CXCR3 (Supplemental Figure 2B, Supplemental Figure 3B, and Supplemental Table 3). Each cluster was then annotated as CD56^bright^, CD56^dim^, CD56^–^, or NKG2C^+^ memory NK cells (Figure 2A, Supplemental Figure 2B, and Supplemental Table 6).

No significant differences were recorded for any of the major NK cell populations between young people with and without LC (Figure 2A). Pediatric LC was associated with decreased levels of cluster 6 (CD56^dim^ NK cell CD16^+^, NKG2A^–^, NKG2C^–^, NKp46^–^) (Figure 2B) and cluster 15 (CD56^bright^ NK cell CD16^+^, NKG2A^+^, NKG2C^–^, NKp46^+^) (Figure 2C) and with increased levels of cluster 25 (CD56^bright^ NK cell CD16^–^, NKG2A^+^, NKG2C^+^, NKp46^+^) (Figure 2D) and cluster 26 (CD56^bright^ NK cell CD16^–^, NKG2A^+^, NKG2C^–^, NKp46^–^) (Figure 2E).

The differential expression of CD28, PD1, CD25, CD95, and CD8 was analyzed in the different NK cell populations by comparing the level of each marker in individuals with and without LC (Figure 2F). Pediatric LC was associated with higher expression of CD25 and decreased expression of CD8 in all NK cell populations. PD1 and CD95 were also increased in pediatric LC but not in all the NK cell populations.

Taken together, the NK cell compartment in pediatric LC shifted from cytotoxic CD16^+^ subsets toward CD16^–^ NK cells, suggesting a dysregulated profile with increased activation but impaired maturation and function.

### T cells are more frequently activated and exhausted but dysregulated in pediatric LC.

We identified 37 clusters in the T cell population using the following markers: TCRVd2, CD4, CD8, CD45RA, CCR7, CD95, CD27, CD28, CCR6, CXCR3, CD25, and CD127 (Supplemental Figure 2C, Supplemental Figure 3C, and Supplemental Table 4). Each cluster was annotated as a γδ T cell, CD4^+^ T cell, CD8^+^ T cell, double-positive T cell, or double-negative T cell (Supplemental Figure 2C). Then, CD4^+^ and CD8^+^ T cell clusters were assigned to the memory, naive, and regulatory subpopulations through expression of the markers CD45RA, CCR7, CD27, CD28, CD95, CD25, and CD127 (Supplemental Table 6).

**Figure 3:**
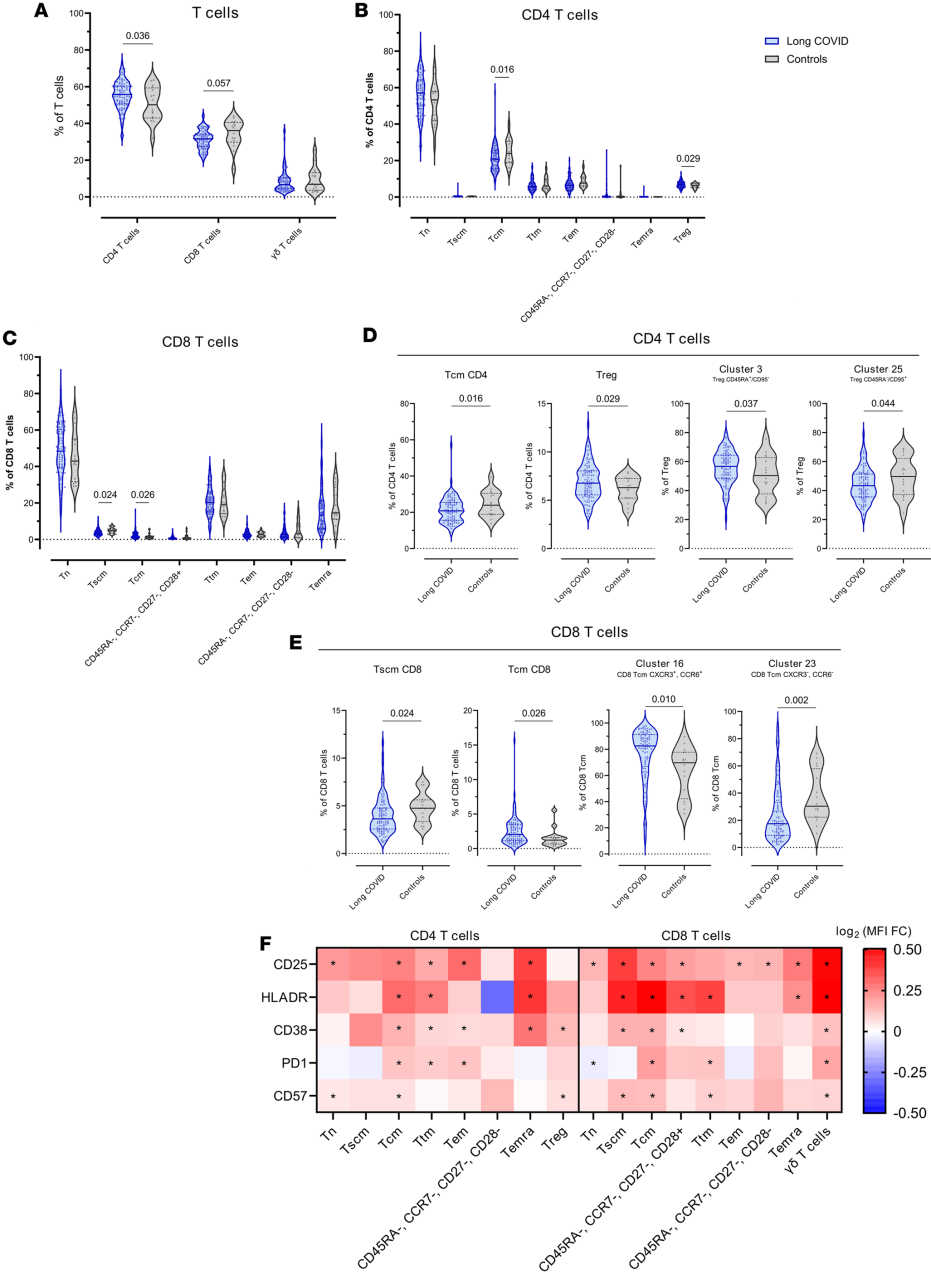
T cell compartment in children and young people with and without LC. (**A**) Frequency of CD4^+^ (*P* = 0.036, FDR = 0.087), CD8^+^ (*P* = 0.057, FDR = 0.087), and γδ T cells (*P* nonsignificant) with respect to total T cells in the LC and control cohorts. (**B**) Frequency of CD4^+^ subpopulations with respect to total CD4^+^ T cells in the LC and control cohorts (CD4^+^ T_CM_ *P* = 0.016, FDR = 0.118; T_reg_ *P* = 0.029, FDR = 0.118; other *P* nonsignificant). (**C**) Frequency of CD8^+^ subpopulations with respect to total CD8^+^ T cells in the LC and control cohorts (CD8^+^ T_SCM_ *P* = 0.024, FDR = 0.118; CD8^+^ T_CM_ *P* = 0.026, FDR = 0.118; other *P* nonsignificant). (**D**) From left to right: frequency of T_CM_ CD4^+^ (*P* = 0.016, FDR = 0.118), frequency of T_reg_ (*P* = 0.029, FDR = 0.118) with respect to total CD4^+^ T cells, frequency of cluster 3 (*P* = 0.037, FDR = 0.274) and frequency of cluster 25 (*P* = 0.044, FDR = 0.274) with respect to total T_reg_ in the LC and control cohorts. (**E**) From left to right: frequency of T_SCM_ CD8^+^ (*P* = 0.024, FDR = 0.118), frequency of T_CM_ CD8^+^ (*P* = 0.026, FDR = 0.118) with respect to total CD8^+^ T cells, frequency of cluster 16 (*P* = 0.010, FDR = 0.126), and frequency of cluster 23 (*P* = 0.002, FDR = 0.058) with respect to total T_CM_ CD8^+^ among the LC and control cohorts. (**F**) MFI fold-change (FC) of CD25, HLADR, PD1, CD38, and CD57 between the LC and control cohorts in each of the different T cell populations. *P < 0.05, FDR < 0.05. The frequency of each cluster and the MFI of the different markers was compared between the LC and control cohorts using linear regression models adjusted for SARS-CoV-2 antigen exposure, sex, and age as covariates. In all analyses, LC group, *n* = 97; control group, *n* = 16. Each dot represents an individual, and median and IQR values are indicated. *P* values less than 0.05 were considered statistically significant and adjusted for multiple testing using FDR.

Pediatric LC was associated with a significant increase in CD4^+^ T cells; CD8^+^ T cells were lower but not significantly different (Figure 3A). Moreover, the CD4/CD8 ratio was higher in the LC group than in the control group, although it did not reach statistical significance (data not shown).

In the CD4^+^ T cell subpopulations, pediatric LC was associated with a decrease in central memory CD4^+^ T cells (CD4^+^ T_CM_) and an increase in regulatory CD4^+^ T cells (T_reg_) (Figure 3, B and D). Moreover, the 2 clusters that formed the T_reg_ population were dysregulated in pediatric LC, with an expansion of cluster 3 (naive T_reg_ CD45RA^+^, CD95^–^) and a decreased proportion of cluster 25 (memory/activated T_reg_ CD45RA^–^, CD95^+^) (Figure 3D).

In the CD8^+^ T cell compartment, pediatric LC was associated with a decrease in stem cell–like memory CD8^+^ T cells (CD8^+^ T_SCM_) and increased central memory CD8^+^ T cells (CD8^+^ T_CM_) (Figure 3, C and E). Among all the clusters, the 2 clusters that formed the CD8^+^ T_CM_ population were dysregulated in the LC group, with increased proportions of cluster 16 (CD8^+^ T_CM_ CXCR3^+^, CCR6^+^) and decreased proportions of cluster 23 (CD8^+^ T_CM_ CXCR3^–^, CCR6^–^) (Figure 3E).

Finally, the differential expression of CD25, HLADR, CD38, PD1, and CD57 was analyzed in the various T cell populations between individuals with and without LC (Figure 3F). Pediatric LC was associated with higher expression of activation (HLA-DR, CD38, CD25) and senescence/exhaustion (CD57, PD-1) markers, particularly in the CD4^+^ T_CM_, CD8^+^ T_CM_, and γδ T cell populations.

In summary, pediatric LC was associated with dysregulation of the T cell compartment driven by decreased CD4^+^ T_CM_ and CD8^+^ T_SCM_ and increased T_reg_ and CD8^+^ T_CM_. Moreover, patients with LC had higher levels of T cell activation characterized by a higher expression of CD25, HLADR, CD38, PD1, and CD57.

### Impaired humoral response against SARS-CoV-2 in pediatric LC.

**Figure 4:**
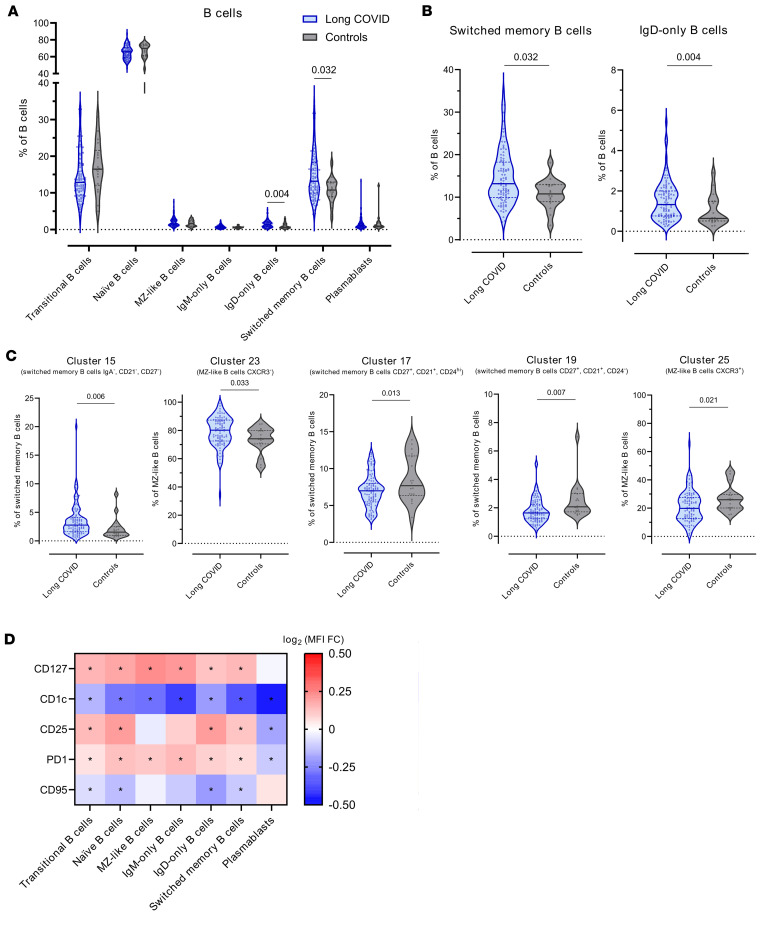
B cell compartment in children and young people with and without LC. (**A**) Frequency of major B cell populations with respect to total B cells among the LC and control cohorts (IgD-only B cells *P* = 0.004, FDR = 0.029; switched memory B cells *P* = 0.032, FDR = 0.110; other *P* = nonsignificant). (**B**) Frequency of switched memory (*P* = 0.032, FDR = 0.110) and IgD-only B cells (*P* = 0.004, FDR = 0.029) with respect to total B cells in the LC and control cohorts. (**C**) From left to right: frequency of cluster 15 (*P* = 0.006, FDR = 0.115) with respect to total switched memory B cells, frequency of cluster 23 (*P* = 0.033, FDR = 0.202) with respect to total MZ-like B cells, frequency of cluster 17 (*P* = 0.013, FDR = 0.127) with respect to total switched memory B cells, frequency of cluster 19 (*P* = 0.007, FDR = 0.115) with respect to total switched memory B cells, and frequency of cluster 25 (*P* = 0.021, FDR = 0.162) with respect to total MZ-like B cells in the LC and control cohorts. (**D**) MFI fold-change (FC) of CD127, CD1c, CD25, PD1, and CD95 between LC and control cohorts in each of the B cell populations. *P < 0.05, FDR < 0.05. The frequency of each cluster and the MFI of the different markers was compared between the LC and control cohorts using linear regression models adjusted for SARS-CoV-2 antigen exposure, sex, and age as covariates. In all analyses, LC group, *n* = 93; control group, *n* = 18. Each dot represents an individual, and median and IQR values are indicated. *P* values less than 0.05 were considered statistically significant and adjusted for multiple testing using FDR.

We identified 31 clusters in the B cell population using the following markers: IgD, IgM, IgA, CD27, CD21, CD38, CD24, CD11c, CCR7, CCR6, CXCR5, and CXCR3 (Supplemental Figure 2D, Supplemental Figure 3D, and Supplemental Table 5). Each cluster was annotated as transitional B cells, naive B cells, marginal zone–like (MZ-like) B cells, IgM-only B cells, IgD-only B cells, switched memory B cells, and plasmablasts (Figure 4A, Supplemental Figure 2D, and Supplemental Table 6).

Pediatric LC was associated with a higher frequency of switched memory and IgD-only B cells (Figure 4B). Specifically, we observed an increase in the frequency of switched memory B cells from cluster 15 (IgA^–^CD27^–^CD21^–^), which lacked the expression of CD27 and CD21, at the expense of the CD27^+^ switched memory B cell subsets (cluster 17 [switched memory B cells CD27^+^CD21^+^CD24^hi^] and cluster 19 [switched memory B cells CD27^+^CD21^+^CD24^-^]) (Figure 4C). Although no differences in the size of the MZ-like B cell compartment were detected, the frequency of MZ-like B cells expressing CXCR3 (cluster 25) was lower in pediatric LC (Figure 4C).

When the expression of CD127, CD1c, CD25, PD1, and CD95 was analyzed in individuals with and without LC, pediatric LC was associated with decreased expression of CD1c in all B cell populations. Interestingly, PD1 expression was increased in all B cell populations except plasmablasts, where it decreased. Similarly, CD25 was increased in transitional, naive, IgD-only, and switched memory B cells but decreased in plasmablasts (Figure 4D).

**Figure 5:**
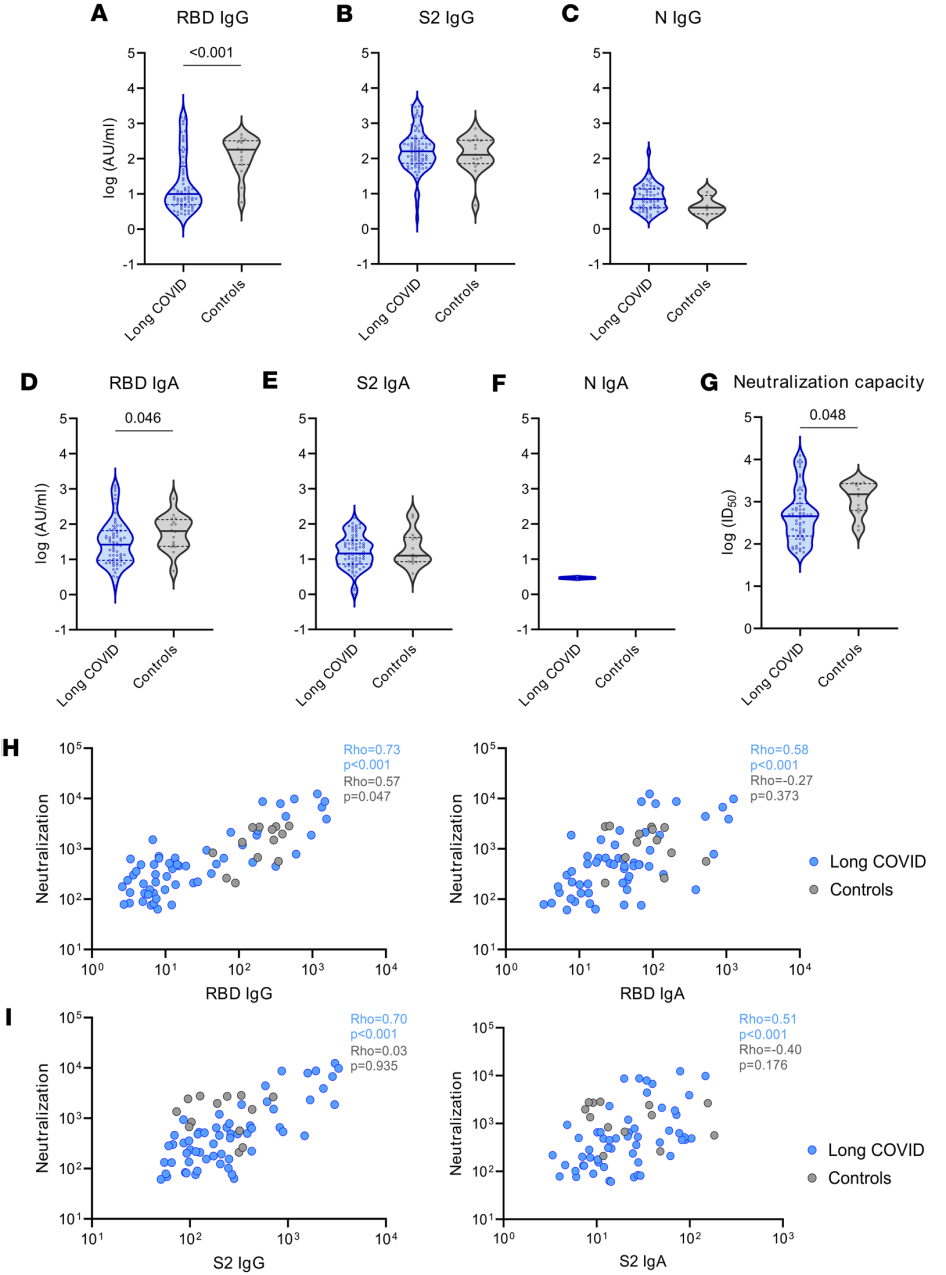
SARS-CoV-2 antibody response in children and young people with and without LC. (**A**) Anti-RBD IgG antibody levels (*P* < 0.001) in responders in log (AU/mL). (**B**) Anti-S2 IgG antibody levels (*P* nonsignificant) in responders in log (AU/mL). (**C**) Anti-N IgG antibody levels (*P* nonsignificant) in responders in log (AU/mL). (**D**) Anti-RBD IgA antibody levels (*P* = 0.046) in responders in log (AU/mL). (**E**) Anti-S2 IgA antibody levels (*P* nonsignificant) in responders in log (AU/mL). (**F**) Anti-N IgA antibody levels (*P* nonsignificant) in responders in log (AU/mL). (**G**) SARS-CoV-2 neutralizing capacity (*P* = 0.048) of responders in log ID_50_ in the LC and control cohorts. (**H**) From left to right: correlation between neutralizing capacity (ID_50_) and anti-RBD IgG antibodies (AU/mL) (LC cohort, anti-RBD IgG/neutralizing capacity, *P* < 0.001, ρ = 0.73; control cohort, anti-RBD IgG/neutralizing capacity, *P* = 0.047, ρ = 0.57), correlation between neutralizing capacity (ID_50_) and anti-RBD IgA antibodies (AU/mL) (LC cohort, anti-RBD IgA/neutralizing capacity, *P* < 0.001, ρ = 0.58; control cohort, anti-RBD IgA/neutralizing capacity, *P* nonsignificant, ρ = –0.27) (**I**) From left to right: correlation between neutralizing capacity (ID_50_) and anti-S2 IgG antibodies (AU/mL) (LC cohort, anti-S2 IgG/neutralizing capacity, *P* < 0.001, ρ = 0.70; control cohort, anti-S2 IgG/neutralizing capacity, nonsignificant, ρ = 0.03), correlation between neutralizing capacity (ID_50_) and anti-S2 IgA antibodies (AU/mL) (LC cohort, anti-S2 IgA/neutralizing capacity, *P* < 0.001, ρ = 0.51; control cohort, anti-S2 IgA/neutralizing capacity, *P* nonsignificant, ρ = –0.40) in the LC and control cohorts. Antibody levels and neutralizing capacity were compared between the LC and control cohorts using linear regression models adjusted for SARS-CoV-2 antigen exposure, sex, and age as covariates. Each dot represents an individual, and median and IQR values are indicated. *P* values less than 0.05 were considered statistically significant.

These results prompted us to check specific antibody levels against SARS-CoV-2 in individuals with and without LC. For that purpose, we used ELISA to quantify plasma levels of IgG and IgA antibodies against SARS-CoV-2 antigens (spike S2, receptor-binding domain [RBD], and nucleocapsid [N].). Pediatric LC was associated with lower levels of anti-RBD IgG and IgA antibodies (Figure 5, A and D). However, we observed no significant differences in anti-S2 IgG and IgA and anti-N IgG antibody levels between the 2 groups (Figure 5, B, C, E, and F). As expected, patients with 2 exposures (vaccinated after acute infection) had significantly higher levels of anti-S2 and anti-RBD antibodies than patients with 1 exposure (either infection or vaccination) (Supplemental Table 7).

To determine whether neutralizing capacity was impaired owing to lower anti-RBD response, we quantified the level of neutralization using a luciferase-reporter lentiviral pseudovirus assay. Pediatric LC was associated with lower levels of neutralizing antibodies against the D614G variant (Figure 5G). However, these levels increased in patients with LC after 2 exposures, as expected (Supplemental Table 7). Moreover, in patients with LC, levels of neutralizing antibodies correlated positively with levels of both anti-RBD and anti-S2 IgG and IgA antibodies (Figure 5, H and I). For the control group, only anti-RBD IgG levels correlated with neutralizing capacity (Figure 5H).

A critical factor to consider when analyzing antibody levels is the time since the most recent antigen exposure (either through vaccination or natural infection), as antibody levels typically diminish over time. Sample collection in the control group was closer to the most recent antigen exposure (median 11.6 weeks; IQR [8.1–17.3]) than in the LC group (median 20.3 weeks; IQR [13.9–34.7]) (Supplemental Figure 4A). However, despite these differences, no correlation was observed between anti-RBD–specific IgG/IgA levels and the time since the last exposure in either the LC or the control group with a single-exposure event (Supplemental Figure 4B). Antibody levels and neutralizing capacity in pediatric patients with LC remained significantly lower when the sample time point of patients with LC was restricted to fewer than 17 weeks since the last antigen exposure to fit within the IQR of the control group, demonstrating that the time from the most recent antigen exposure did not affect the differences observed between the LC group and the control group (Supplemental Figure 4, C–E).

Given that we observed reduced specific antibody levels and neutralizing capacity against SARS-CoV-2 in pediatric LC, we aimed to investigate potential viral persistence in the plasma of patients with LC. To this end, we employed the MSD S-plex assay to quantify antigen persistence (spike and nucleocapsid) and droplet digital PCR to detect total, genomic, and subgenomic SARS-CoV-2 RNA in plasma. Pediatric LC was occasionally associated with antigen persistence for both spike and nucleocapsid, with 6.1% and 9.1% of samples testing positive, respectively, whereas none of the controls were positive for either protein (Supplemental Figure 6A). Moreover, time since infection did not correlate with antigen levels among those who tested positive (Supplemental Figure 6B). Regarding SARS-CoV-2 RNA, 5 LC participants were positive (3 for 3′UTR [total RNA] and 2 for subgenomic N [Nsg]), and 2 controls were positive for Nsg copies (Supplemental Table 8). These findings are consistent with the known false-positive rate of droplet digital PCR (19, 20), rendering the results unreliable as evidence of viral persistence.

Overall, pediatric LC was characterized by dysregulation of the B cell compartment and an impaired anti-RBD humoral response associated with decreased neutralizing activity against SARS-CoV-2 and potential antigen viral persistence.

### Decreased CCR6 expression in monocytes distinguishes patients with LC from controls.

**Figure 6:**
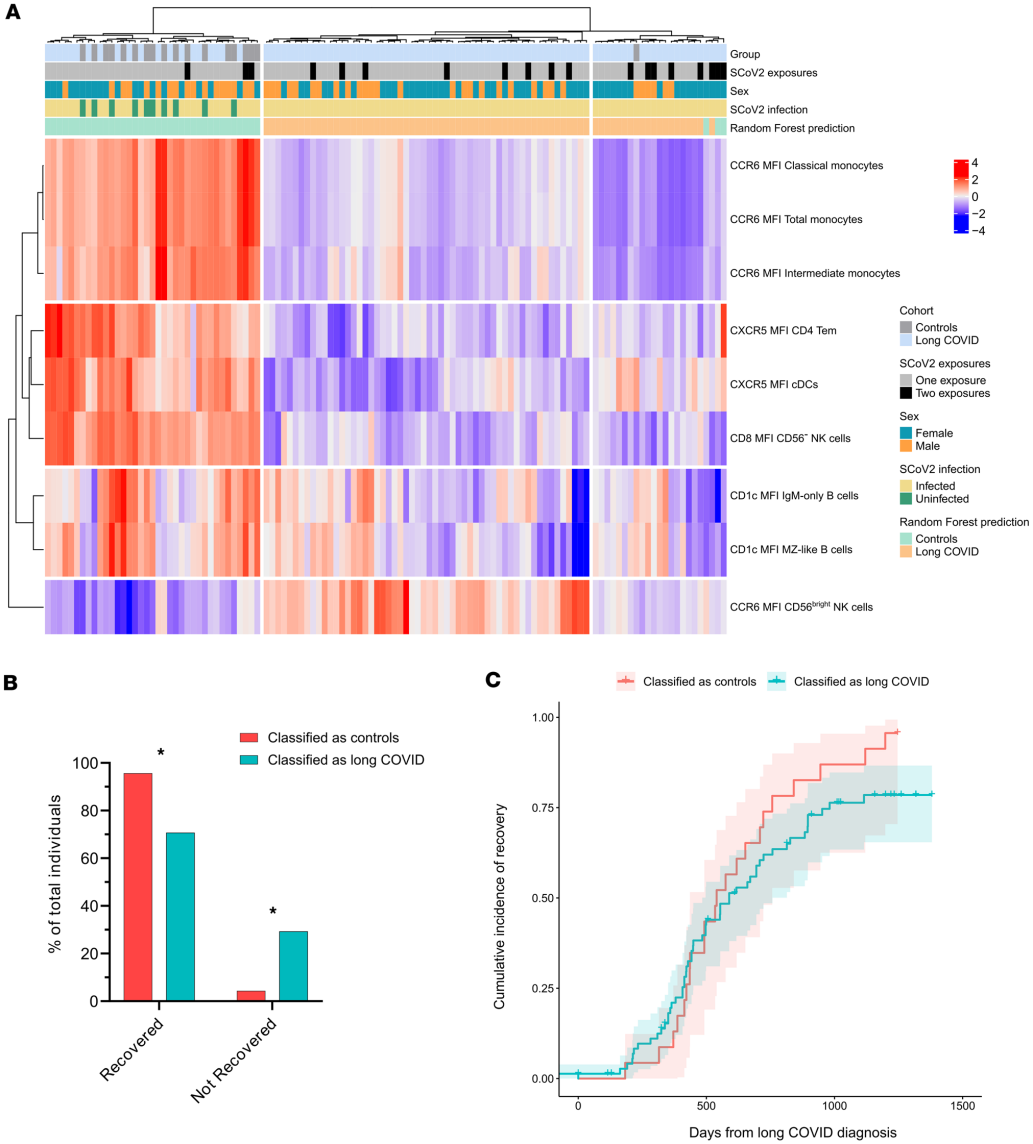
Random forest model classifies patients with LC and controls with 79.3% accuracy. (**A**) Heatmap representing the 9 most important features that distinguish patients with LC from controls in the random forest model (from most important CCR6 MFI classical monocytes to least important CCR6 MFI CD56^bright^ NK cells) (LC group, *n* = 98; control group, *n* = 18). (**B**) Percentage of recovered individuals who were classified as controls or LC by the random forest model (classified as controls *n* = 23; classified as LC *n* = 75; *P = 0.012, Fisher’s exact test). (**C**) Kaplan-Meier curves showing the proportion of recovered patients with LC over time in individuals classified as controls or LC by the random forest model (classified as control group, *n* = 23; classified as LC group, *n* = 75; *P* nonsignificant).

To identify immunophenotypic features distinguishing patients with LC from controls, we used random forest–based classification. The random forest model achieved an overall accuracy of 79.3% in differentiating LC cases from controls (Supplemental Figure 7). Among all the parameters analyzed, the 9 most influential were the following: CCR6 expression in classical, total, and intermediate monocytes; CXCR5 expression in effector memory CD4+ T cells (CD4+ T_EM_) and cDCs.”; CD8 expression in CD56^–^ NK cells; CD1c expression in MZ-like and IgM-only B cells; and CCR6 expression in CD56^bright^ NK cells (Figure 6A). Notably, CCR6 expression on classical, total, and intermediate monocytes was the most significant factor for distinguishing patients with LC from control individuals. Moreover, only CCR6 expression in CD56^bright^ NK cells and CD8 expression in CD56^–^ NK cells showed weak correlation with the time since last antigen exposure in the LC group (ρ = 0.267 and –0.256, respectively) (Supplemental Table 9). When comparing the 9 most important features between infected and uninfected (vaccinated) controls, only CCR6 expression in CD56^bright^ NK cells differed significantly between these groups (Supplemental Figure 8A and Supplemental Table 10). Although infected controls had higher expression than uninfected (vaccinated) controls, the LC group demonstrated significantly increased expression compared with both groups. Additionally, among the cellular populations and clusters that differed significantly between the LC and control groups, infected and uninfected (vaccinated) controls showed significant differences only in B cell clusters 15 and 19 (Supplemental Figure 8, B and C).

Finally, we observed that individuals diagnosed with LC who were misclassified as controls by the random forest model exhibited a higher rate of recovery than those classified correctly (96.0% vs. 71.0%, *P* = 0.012) (Figure 6B). However, there were no significant differences in the time from diagnosis to recovery between the 2 groups (Figure 6C). Moreover, no other clinical parameter — such as number or duration of symptoms, or time since acute infection — correlated with LC patient classification by the random forest model.

## Discussion

Elucidating LC’s pathophysiology and identifying reliable biomarkers remain challenging in both children and adults. In this study, the comprehensive analyses of PBMCs and plasma from young individuals with and without LC revealed significant immunological differences.

Immunophenotyping of major immune cell populations (myeloid, NK, T, and B cells) revealed significant differences between young individuals with and without LC. Notably, pediatric LC was associated with an increased proportion of DCs relative to monocytes. Although the overall proportion of monocytes was reduced, there was a specific increase in nonclassical monocytes, cells implicated in chronic inflammation and autoimmunity, and previously reported as elevated in adults with LC (21, 22). Moreover, the pediatric LC group showed increased pDCs and decreased cDCs compared with the control group. pDCs are the primary producers of type I IFNs, with aberrant or delayed response linked to tissue damage in COVID-19 (23), while cDCs primarily initiate adaptive immune responses (24). Notably, the cluster 7 DC subset (cDC, CD14^+^, CD1c^+^), resembling the DC3 subset that effectively stimulates T cells and promotes CD8 resident memory differentiation (25), was significantly reduced in pediatric LC. Across all major DCs and monocyte populations, CCR7 and CCR6 expression was decreased, as was CXCR5 and CD169 in most populations. Since interaction of CCL20 with CCR6 mediates recruitment of DCs and monocytes to inflamed and mucosal tissues (26), its reduction may impair antigen-presenting cell migration and local immune responses. Similarly, lower CCR7 expression could hinder antigen-presenting cell migration to lymph nodes, weakening T and B cell activation. Overall, these changes may disrupt antigen processing and maturation, leading to suboptimal adaptive immune responses, findings consistent with reports of SARS-CoV-2 persistence in tissues such as the gut (13, 27).

No significant differences were observed in the major NK cell populations (CD56^bright^, CD56^dim^, CD56^–^) between individuals with and without LC. However, 4 NK cell clusters were dysregulated in pediatric LC: those that decreased were CD16^+^, whereas those that increased lacked CD16. CD16 (FcγRIII) is essential for NK cell–mediated antibody-dependent cellular cytotoxicity (28). Additionally, pediatric LC was characterized by higher CD25 expression and lower CD8 expression across all NK cell subsets. CD25 upregulation reflects NK cell activation and enhances cytotoxic function (29), whereas CD8, though primarily a T cell marker, is also found on some NK cells (30) and is associated with greater functional activity, as seen in people living with HIV-1 (31).

Pediatric LC was marked by an increased frequency of CD4^+^ T cells and elevated T_reg_ levels, alongside reduced CD4^+^ T_CM_. These features have also been reported in adults with LC (22, 32). Analysis of the T_reg_ population showed 2 distinct clusters in pediatric LC: higher levels of naive CD45RA^+^ T_reg_ and lower levels of memory-like CD45RA^–^ T_reg_ (33), the latter being predominant among activated Foxp3^+^ cells (34). In the CD8^+^ T cell compartment, pediatric LC was characterized by decreased CD8^+^ T_SCM_ and increased CD8^+^ T_CM_, which are critical for long-term immune protection due to their proliferative potential and ability to generate effector cells upon antigen reexposure (35, 36). Recent evidence indicates that T_CM_, due to their location in secondary lymph nodes and superior proliferative capacity, play a more central role than T_EM_ in controlling systemic infections (37). Additionally, patients with LC had more CD8^+^ T_CM_ expressing CXCR3 and CCR6, a pattern that aligns with traits observed in chronic viral infections such as HIV-1 (38). Finally, pediatric LC was associated with heightened activation and features of senescence/exhaustion in CD4^+^, CD8^+^, and γδ T cell subsets, evidenced by increased expression of CD25, HLADR, CD38, PD1, and CD57.

B cell immunophenotyping revealed that patients with LC had higher frequencies of IgD-only B cells and total switched memory B cells compared with controls. Although a previous study found no significant differences in the B cell compartment between children with LC and recovered individuals (39), their analysis used a more limited set of markers, whereas our profiling was broader. IgD-only B cells, known to be enriched for autoreactivity (40), were increased in LC, though the functional importance of these subsets in LC remains unclear and warrants further investigation.

Despite the overall increase in switched memory B cells, we observed dysregulation within the IgG^+^ memory B cell compartment, specifically increased IgA^–^CD21^–^CD27^–^ (cluster 15) and decreased IgA^–^CD21^+^CD27^+^CD24^hi^ (cluster 17) and IgA^-^CD21^+^CD27^+^CD24^–^ (cluster 19) switched memory B cells. Cluster 15 cells, which often express inhibitory receptors like FcRL4 and FcRL5, displayed an “exhausted-like” phenotype common in chronic viral infections such as HIV-1 (41). Although FcRL4 was not directly measured here, FcRL4^+^ B cells are less likely to differentiate into antibody-secreting plasma cells (42). Notably, CD21^–^CD27^–^ memory B cells can originate from extrafollicular humoral responses or primary germinal center reactions (43), suggesting impaired germinal center maturation in young patients with LC. In contrast, the CD21^+^CD27^+^ subsets (clusters 17 and 19) are typically associated with higher proliferation, increased somatic hypermutation, and robust antibody production upon antigen reexposure (44). The expansion of CD21^–^CD27^–^ and contraction of CD21^+^CD27^+^ memory B cells, characteristic of acute COVID-19, is typically resolved after recovery (45); its persistence in pediatric LC suggests sustained humoral immune dysfunction.

Compared with the control group, the LC group had a higher frequency of MZ-like B cells lacking CXCR3 and a lower frequency of CXCR3^+^ MZ-like B cells, although the total proportion of MZ-like B cells was unchanged. The primary distinction between these clusters was CXCR3 expression, a chemokine receptor critical for directing activated immune cells to sites of inflammation and implicated in murine models of rheumatoid arthritis (46). CXCR3^+^ MZ-like B cells are associated with T-bet expression and IL-10 production (46), but the role of CXCR3 in human MZ-like B cell trafficking remains unclear and warrants further research in pediatric LC. Lastly, pediatric LC showed increased CD127, CD25, and PD1 expression across all B cell populations except plasmablasts, which had reduced CD25 and PD1, suggesting lower activation and potentially decreased antibody production. CD1c expression was also diminished in B cells from the LC group; since reduced CD1c in B cells activated via the CD40/CD40L pathway is linked to impaired antigen presentation (47), this suggests possible deficiencies in B cell–mediated immune responses in this group.

Our analysis of the B cell compartment revealed that, while the proportion of antibody responders and nonresponders was similar between groups, individuals with LC had significantly lower levels of anti-RBD IgG and IgA antibodies. Conversely, anti-S2 and anti-N IgG and IgA levels did not differ, indicating that the LC group exhibited a selective reduction in humoral responses targeting the RBD region of the SARS-CoV-2 spike protein. This finding contrasts with adult LC studies, where elevated anti-RBD and other spike antibody responses have been reported (22, 32, 48); however, comparisons are limited due to differences in vaccination status, as most adults studied were vaccinated, whereas most children and young people in our cohort were unvaccinated at the time of sample collection. Consistent with the reduced anti-RBD antibody levels, neutralization assays showed significantly lower SARS-CoV-2–neutralizing capacity in the LC group. Since the RBD is a primary target of neutralizing antibodies (49), this reduction is expected. Reduced neutralization capacity in LC has also been observed in recent UK studies (50). The lower anti-RBD antibody levels and neutralization capacity in pediatric LC may limit viral clearance during acute infection, potentially allowing for viral persistence in tissue reservoirs, a hypothesis supported by research linking low acute-phase antibody titers to increased LC risk (51–53). However, in our cohort, fewer than 10% of participants tested positive for SARS-CoV-2 antigens, with only rare cases showing concurrent positivity, suggesting a limited role for plasma viral persistence in pediatric LC. Moreover, we previously analyzed extracellular vesicles isolated from plasma of patients with severe COVID-19 and did not detect any SARS-CoV-2 protein, supporting our current findings (54). Future studies should investigate viral persistence in tissue samples from pediatric LC cases, as has been done in adults (13, 27).

A key limitation of our study is the relatively small size and imbalanced control group, which was about one-fifth the size of the LC group. This imbalance stemmed from recruitment challenges, as only children and adolescents without LC, inflammatory diseases, or acute infections at sampling were eligible. As a result, we relied on healthy pediatric volunteers whose parents or guardians consented to blood collection. Additionally, pediatric COVID-19 vaccination had commenced during recruitment, making it impractical to exclude participants based on vaccination status. Consequently, most controls (72.2%) were vaccinated prior to sampling, compared with only 16.2% of the LC group. While this discrepancy could confound the observed lower anti-RBD IgG and IgA levels observed in the LC group, it is unlikely the sole explanation. If vaccination were primarily responsible, we would expect similar reductions in anti-S2 antibodies, which were not observed. Moreover, recent studies have reported no significant differences in total IgG levels between vaccinated pediatric individuals and those with natural infection (55), supporting the idea that reduced anti-RBD antibodies in LC are not solely attributable to vaccination. Previous immunophenotyping data also revealed no major differences in SARS-CoV-2–specific T and B cell populations between previously infected and vaccinated individuals, suggesting that vaccination alone does not substantially alter these subsets (56). Furthermore, among controls, only CCR6 expression in CD56^bright^ NK cells and 2 B cell clusters (15 and 19) differed between uninfected (vaccinated) and infected individuals, with the lowest CCR6 expression in uninfected (vaccinated) controls. Importantly, the 9 key features distinguishing the LC group from controls were unaffected by vaccination status, and all analyses were adjusted for SARS-CoV-2 antigen exposure to account for its influence on antibody responses and immune cell composition.

Another limitation involves the timing of sample collection, as control samples were obtained significantly closer to their most recent antigen exposure than those from the LC group. Nevertheless, children and young people with LC consistently showed markedly lower anti-RBD IgG and IgA antibody levels, regardless of the interval since their last antigen exposure. Moreover, SARS-CoV-2 neutralization capacity remains stable for more than 6 months after exposure (57), making it unlikely that differences in neutralization potential are attributable to sampling time. Of the 9 key immunophenotyping features distinguishing the LC group from the control group, only CCR6 expression in CD56^bright^ NK cells and CD8 expression in CD56^–^ NK cells showed a weak correlation with time since last antigen exposure in the LC group, indicating that sampling timing had minimal impact on the random forest model’s most important distinguishing features.

Finally, using random forest classification, we identified reduced CCR6 expression in monocytes as the strongest discriminator between the LC and control groups. Remarkably, our model achieved nearly 80% accuracy with just 9 immunophenotyping variables. Additionally, LC cases misclassified as controls showed higher clinical recovery rates, suggesting that normalization of immune profiles may be linked to symptom resolution.

In summary, our study offers a detailed characterization of innate and adaptive immune responses in children and young people with and without LC. The findings suggest that impaired innate immunity — particularly reduced antigen-presenting cell migration to secondary lymphoid and inflamed tissues — may limit CD4^+^ T cell responses, leading to suboptimal germinal center activity, lower antibody production, and decreased SARS-CoV-2 neutralization. Additionally, the heightened activation of T, B, and NK cells likely represents a compensatory response to the limited CD4^+^ T cell pool, with overactivation serving to maintain immune homeostasis.

## Methods

### Sex as a biological variable.

Our study examined male and female pediatric individuals with and without LC. Sex and age were used to correct any statistical analysis as the LC group was predominately of female sex.

### Study participants, sample processing, and data collection.

PediaCOVID cohort participants were recruited between January 2021 and February 2022 at University Hospital Germans Trias i Pujol (Badalona, Spain). The LC group included individuals younger than 18 years diagnosed with SARS-CoV-2 infection (PCR, antigen test, serology, cellular immunity, and/or clinical evaluation) and with at least 12 weeks of persistent COVID-19 symptoms after acute COVID-19 (5). The control group comprised individuals younger than 18 years without LC, active SARS-CoV-2 infection, or immune abnormalities at the time of sample collection who had previously been diagnosed or not with SARS-CoV-2 infection (PCR, antigen test, serology, and/or clinical evaluation). Blood samples were prospectively collected from the pediaCOVID cohort (University Hospital Germans Trias i Pujol ethics committee, ID PI-21-029), and plasma and PBMCs were isolated using Ficoll-Paque and cryopreserved for further studies. We analyzed 99 samples from the LC group and 18 samples from children and young people without LC (control group). All the participants had mild or asymptomatic acute infection, were not hospitalized, and did not receive any treatment or intervention during acute illness. To account for potential SARS-CoV-2 vaccination when analyzing the immune response in both the LC and control groups, we further stratified participants into those who experienced 2 exposures to viral antigens (infection plus 1 vaccine dose) and those who had a single exposure to viral antigens (either infection or full vaccine regimen).

The data recorded included demographic data, medical and family history, SARS-CoV-2 infection data, symptoms during the acute phase of COVID-19 (in symptomatic cases), and persistent symptoms (e.g., headache, fatigue, brain fog). Children and young people whose SARS-CoV-2 infection was not microbiologically confirmed by PCR, antigen test, serology, or SARS-CoV-2–specific T cell immunity were diagnosed through clinical evaluation (history, symptoms, and examination).

### Multiparametric spectral cytometry immunophenotyping (37-color panel).

PBMCs were stained with the 37-color antibody panel summarized in Supplemental Table 1, fixed with 1% paraformaldehyde (Sigma Aldrich), and analyzed in a Cytek Aurora 5-laser spectral cytometer. Cytometry data were analyzed using FlowJo version 10.10 (BD Life Sciences) and OMIQ (Dotmatics: www.omiq.ai, www.dotmatics.com). A manual gating strategy was followed to export the different immune cell populations in a separate file (myeloid cells, NK cells, T cells, and B cells) (Supplemental Figure 1). The files containing the different immune cell populations were then uploaded and analyzed in the OMIQ platform. The FlowSOM algorithm was used to identify clusters of the different immune cell populations. Myeloid cells were clustered using CD1c, CD11b, CD16, CD11c, HLADR, CD95, CD14, and CD123. NK cells were clustered using CD56, CD16, CD57, NKG2A, NKG2C, NKp46, CCR5, and CXCR3. T cells were clustered using TCRVd2, CD4, CD8, CD45RA, CCR7, CD95, CD27, CD28, CCR6, CXCR3, CD25, and CD127. B cells were clustered using IgD, IgM, IgA, CD27, CD21, CD38, CD24, CD11c, CCR7, CCR6, CXCR5, and CXCR3. Finally, after cluster identification and annotation, dimensionality reduction was performed using uniform manifold approximation and projection (UMAP) with the same markers used for clustering, analyzing 200,000 events per immune cell population (Supplemental Figures 2 and 3).

### Quantification of SARS-CoV-2–specific IgG and IgA.

To determine SARS-CoV-2–specific IgG and IgA titers, MaxiSorp ELISA plates were coated with anti-His tag monoclonal antibody (Thermo Fisher Scientific, MA1-21315) at 2 μg/mL and incubated overnight at 4°C. The following day, plates were washed and blocked with 1% BSA in 1× PBS for 2 hours at room temperature. A half-plate was then incubated with S2, RBD, or nucleocapsid (wild-type [Wuhan] strain; SinoBiological) at 1 μg/mL overnight at 4°C. The other half-plate received antigen-free blocking buffer to determine sample background. Heat-inactivated (incubated at 56°C for 30 minutes) plasma samples were then added to the corresponding antigen-coated and antigen-free wells of the same plate and incubated for 1 hour at room temperature. Next, HRP-conjugated goat anti-human IgG or IgA (Jackson ImmunoResearch 109-036-098 and 109-035-011) was added to appropriate wells for 30 minutes at room temperature. Plates were then developed using o-phenylenediamine dihydrochloride (Sigma-Aldrich, P8412-50TAB). The enzymatic reaction was stopped with 2N H_2_SO_4_. Absorbance was measured using the EnSight Multimode Plate Reader at 492 nm with noise correction at 620 nm. The specific signal was determined by subtracting the absorbance obtained in antigen-free wells from that of the wells coated with SARS-CoV-2 proteins. The results are expressed as arbitrary units per mL (AU/mL).

### Quantification of specific SARS-CoV-2 neutralizing capacity.

Neutralization assays were performed in duplicate as previously described (58). Briefly, 200 median tissue culture infectious dose 200 (TCID50) of pseudovirus was preincubated in Nunc 96-well cell culture plates (Thermo Fisher Scientific), with 3-fold serial dilutions (1/60–1/14,580) of heat-inactivated (incubated at 56°C for 30 minutes) plasma samples for 1 hour at 37°C. Then, 1 × 10^4^ HEK293T/hACE2 cells treated with DEAE-Dextran (Sigma-Aldrich) were added. The results were read after 48 hours using the EnSight Multimode Plate Reader and BriteLite Plus Luciferase reagent (PerkinElmer). The values were normalized, and the ID_50_ was calculated by plotting and fitting all duplicate neutralization values and the log of the plasma sample dilution to a 4-parameter equation in GraphPad Prism 9.0.2. This assay has previously been validated with a replicative viral inhibition assay (59).

### SARS-CoV-2 viral RNA and antigen detection.

Plasma samples from participants were thawed and centrifuged at 1,000*g* for 10 minutes. From the same aliquot, 100 μL were reserved for SARS-CoV-2 antigen detection experiments, and 1 mL for SARS-CoV-2 RNA detection. MSD S-plex assays were used to detect both SARS-CoV-2 spike (K150ADJS) and nucleocapsid (K150ADHS) proteins. Prior to the assay, plasma samples were treated with Triton X-100 (Sigma-Aldrich) at a final concentration of 0.05% and incubated for 30 minutes to inactivate the virus. After inactivation, to dissociate potential immunocomplexes, samples were incubated with 1 M HCl for 10 minutes at room temperature, after which HCl was neutralized with 1.2 M NaOH in 0.5 M HEPES buffer. The assay was then performed according to the manufacturer’s protocol. Values from uninfected controls were used to establish the limit of detection of both spike and nucleocapsid antigens.

For SARS-CoV-2 RNA detection, 1 mL of plasma was ultracentrifuged at 170,000*g* for 30 minutes at 4°C. Then, the supernatant was collected, and RNA was extracted using the KingFisher Flex Purification system with the Maxwell HT Viral TNA kit (Promega). After RNA extraction, SARS-CoV-2 3′UTR (total), N (total), N genomic, and Nsg copies were quantified by droplet digital PCR (QX600, BioRad) (60–62).

### Statistics.

Cell frequency, antibody levels, and neutralizing capacity in each group were compared between the LC and control groups using linear regression models adjusted for SARS-CoV-2 antigen exposure, sex, and age as covariates. For each cluster, data were transformed using either logarithmic or square root methods based on the Box-Cox approach, selecting the transformation that best approximated a normal distribution.

The likelihood ratio test was used to compare nested models with and without the group factor. The same type of regression models, including covariate adjustment, was used to analyze differential expression of CD169, CD38, CCR6, CCR7, CCR5, CXCR3, CXCR5, PD1, CD25, CD95, CD8, HLADR, CD57, CD127, and CD1c, and model-based fold-changes were estimated between groups.

When the LC and control groups were matched by time from last exposure, antibody levels and neutralizing capacity were compared using the Mann-Whitney *U* test (nonparametric test); Spearman’s correlations were used for the correlation analysis.

In order to identify the markers with the highest discriminative capacity between the LC and control groups, a classification model based on random forest analysis was used, incorporating all the results for population frequency, differential expression analyses, and antibody levels. The random forest model was fitted with 5,000 classification trees and tuned to account for class imbalance between the 2 groups. The number of markers selected was determined through multiple selection strategies based on their importance scores, ranging from more to less conservative. The random forest model was refitted using only the selected markers in each case, and the resulting models were compared based on their out-of-bag estimate of error rate. Finally, a heatmap combined with hierarchical clustering was used to visually summarize patterns of marker expression across samples and differences between groups.

To determine whether differences existed between the infected and uninfected (vaccinated) controls, we compared the levels of the cellular populations and clusters that were significantly different between the LC and control groups, as well as the 9 most important features distinguishing the LC group from the control group, using the nonparametric Mann-Whitney *U* test.

A survival analysis was conducted to analyze the potential relationship between the classification made by the random forest model within the LC group (based on immunological markers) and the clinical severity of the disease. This analysis aimed to determine whether LC samples misclassified by the random forest model were associated with different clinical conditions. It focused on the time from the LC diagnosis to medical discharge, estimating survival curves using the Kaplan-Meier method and plotting the cumulative recovery curve accordingly.

*P* values less than 0.05 were considered statistically significant. In all analyses, *P* values were adjusted for multiple testing using the Benjamini-Hochberg FDR method.

The analyses were performed using R version 4.4.1 and GraphPad Prism version 10.2.2 for Windows. The following R packages were used: randomForest (https://CRAN.R-project.org/doc/Rnews/) (63), ComplexHeatmap (64), and survival analysis (https://CRAN.R-project.org/package=survival) (65).

### Study approval.

The study was approved by the University Hospital Germans Trias i Pujol ethics committee, Badalona, Spain (ID PI-21-029). Parents/legal guardians and individuals older than 12 years agreed to collection of their samples and signed the informed consent document.

### Data availability.

Pseudonymized raw data and a data dictionary for the dataset are available from Zenodo data repository (66). To gain access, data requesters will need to sign a data access agreement. For the source data, the Supporting Data Values file — including values for all data points shown in the graphs and the values behind any reported means — is available.

## Author contributors

JIP, JMP, and SML were responsible for the design and execution of the study. AGA, CCA, M. Mendez, CR, and JD were responsible for the clinical data collection, participant recruitment and cohort assembly, and sample collection. MN, M. Massanella, JB, and JC were responsible for the design of the spectral cytometry panel used. JIP and SML were responsible for the spectral cytometry analysis. NPL and JC were responsible for the specific SARS-CoV-2 antibody level analysis. JIP, TP, JB, and BT were responsible for the specific SARS-CoV-2 neutralizing capacity analysis. VU, JIP, and SML were responsible for the statistical analysis. JIP and SML were responsible for writing the manuscript. JIP, SML, and JMP were responsible for the decision to submit the manuscript. NPL, TP, MN, VU, FL, FML, JD, AGA, CCA, M. Mendez, CR, M. Massanella, JB, JC, BT, and JMP provided important critical reviews and feedback on the manuscript. JMP and SML as co-corresponding authors confirm that all authors have read and agreed with the current version of the manuscript. The co-corresponding authors had full access to all the data in the study and had final responsibility for the decision to submit for publication.

## Funding support

The following entities provided funding support.

- Barcelona City Council and the Spanish Ministry of Science and Innovation 22S09391-001.
- University Hospital Germans Trias i Pujol LC2313.
- PhD Joan Oró fellowship 2023 FI-1 00207 from the Catalan Agency of Management of University and Research Grants (to JIP).
- EU Gilead Research Scholars Program in HIV award 2021 and the Catalan Agency of Management of University and Research Grants 2020 BP 00046 (to SML.)
- Juan de la Cierva postdoctoral fellowship FJC2021-047205-I (to NPL).
- Spanish Ministry of Science and Innovation grant RYC2020-028934-I/AEI/10.13039/501100011033.
- European Social Fund “Investing in Your Future.”
- Gilead grant GLD21-00070 (to M. Massanella).
- Spanish Ministry of Science and Innovation grants PID2022-139271OB-I00 and CB21/13/00063 (to JMP).
- Fundació La Marató de TV3 grant 202130-30-31-32 (to JMP).
- “The human genetic and immunological determinants of the clinical manifestations of SARS-CoV-2 infection: Towards personalized medicine” (UNDINE) project, which has received funding under the Horizon Europe Research and Innovation programme (grant agreement 101057100) (to JMP).
- CERCA Programme/Generalitat de Catalunya (2021 SGR 00452) for institutional support.

## Supplementary Material

Supplemental data

Supporting data values

## Figures and Tables

**Table 1:**
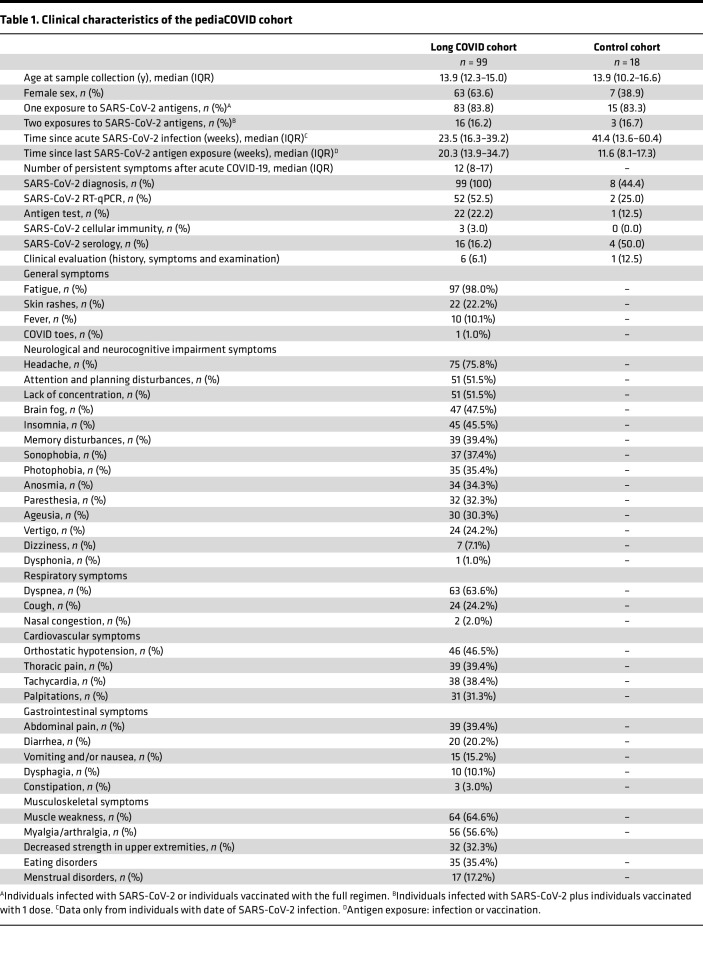
Clinical characteristics of the pediaCOVID cohort

## Version 1 02/19/2026

In-Press Preview

## Version 2 04/08/2026

Electronic publication
