# Supplementary material for: Pediatric long COVID is characterized by myeloid CCR6 suppression and immune dysregulation
Source: JCI Insight. 2026 Feb 19;11(7):e201111. doi: 10.1172/jci.insight.201111 (PMC13134710; doi:10.1172/jci.insight.201111)
Supplement: Supplemental data [file jciinsight-11-201111-s193.pdf]

Pediatric Long COVID Is Characterized by Myeloid CCR6 Suppression and Immune Dysregulation – Supplemental information

Supplemental Table 1: 37-color panel used for spectral cytometry.

Marker	Fluorochrome	Clone
CD19	Spark NIR 685	HIB19
HLADR	BV510	L243
IgD	BV480	IA6-2
IgM	BV570	MHM-88
IgA	FITC	REA1014
CD27	APC	M-T271
CD21	V405	B-ly4
CD38	APC/Fire 810	HIT2
CD24	BUV615	ML5
CD11c	BUV805	B-ly6
CCR7	BV421	G043H7
CCR6	BV711	G034E3
CXCR5	BV750	RF8B2
CXCR3	PE/Cy7	G025H6
CD1c	Alexa Fluor 647	L161
PD-1	BV785	EH12.2H7
CD95	PE/Cy5	DX2
CD25	PE-Alexa Fluor 700	3G10
CD127	APC-R700	HIL-7R-M21
CD3	PerCP	OKT3 (SKT7)
TCR Vδ2	PerCP-Vio	REA771
CD14	Spark Blue 550	63D3
CD4	cFluor 568	SK3
CD8	Pacific Orange	3B5
CD45RA	BUV395	5H9
CD28	BV650	CD28.2
CD57	BV605	C10-1
CCR5	BUV563	2D7
CD56	BUV737	NCAM16.2
CD16	BUV496	3G8
NKG2C	BUV661	134591
NKG2A	PE-Vio615	REA110
NKp46	APC/Fire 750	9E2
CD123	Super Bright 436	6H6
CD11b	BB515	ICRF44
CD169	BB700	7-239
CD137L	PE	5F4

Supplemental Table 2: Cell count, marker annotation and myeloid cell population for 17 clusters

		Marker									
Cluster	Cell count	CD14	CD16	CD11c	CD123	CD1c	CD11b	HLADR	CD95	Myeloid cell population 1	Myeloid cell population 2
1	342,575	+	+	+	-	-	+	+	+	Monocytes	Intermediate monocytes
2	7,583,745	+	-	+	-	-	+	+	+	Monocytes	Classical monocytes
3	192,759	-	+	+	-	-	-	+	+	Monocytes	Non-classical monocytes
4	8,905	-	+	-	-	-	low	low	+	Monocytes	Non-classical monocytes
5	33,642	-	+	-	-	-	low	-	+	Monocytes	Non-classical monocytes
6	452,565	-	-	+	-	+	-	+	+	Dendritic cells	cDCs
7	68,514	+	-	+	-	+	-/low	+	+	Dendritic cells	cDCs
8	273,434	+	-	+	-	-	low	+	+	Monocytes	Classical monocytes
9	69,908	-	-	-	-	-	-	low	+	Dendritic cells	CD123- CD11c- DCs
10	74,789	+	-	low	-	-	low	low	+	Monocytes	Classical monocytes
11	11,384	+	-	+	+	-	+	+	+	Monocytes	Classical monocytes
12	1,441,128	+	-	+	-	-	+	low	+	Monocytes	Classical monocytes
13	649,848	+	-	+	-	-	+	-	+	Monocytes	Classical monocytes
14	653,258	-	-	-	+	-	-	+	-	Dendritic cells	pDCs
15	72,292	-	-	+	-	-	-	+	-	Dendritic cells	cDCs
16	354,045	-	-	-	-	-	-	+	-	Dendritic cells	CD123- CD11c- DCs
17	40,095	+	-	-	-	-	+	+	-/+	Monocytes	Classical monocytes

Supplemental Table 3: Cell count, marker annotation and NK-cell population for 33 clusters

Cluster	Cell count	Marker								NK-cell population
		CD56	CD16	CD57	NKG2C	NKG2A	NKp46	CCR5	CXCR3	
1	2,306,493	dim	+	-	-	+	-	-	-	CD56dim
2	46,356	-	+	-	-	-	-	-	+	CD56-
3	56,548	dim	+	-	-	-	-	-	+	CD56dim
4	393,957	-	+	-	-	-	-	-	-	CD56-
5	3,284,317	dim	+	-	-	-	-	-	-	CD56dim
6	18,331	dim	+	-	-	-	-	+	+	CD56dim
7	350,004	dim	+	-	-	+	-	-	+	CD56dim
8	33,772	dim	-	-	-	+	-	-	+	CD56dim
9	99,504	dim	-	-	-	+	+	-	+	CD56dim
10	187,417	dim	+	-	-	+	+	-	+	CD56dim
11	25,002	-	+	-	-	-	+	-	low	CD56-
12	150,315	dim	+	-	-	-	+	-	+	CD56dim
13	379,804	dim	+	+	-	-	-	-	-	CD56dim
14	85,325	dim	+	-	-	+	+	+	+	CD56dim
15	211,698	bright	+	-	-	+	+	-	+	CD56bright
16	45,591	bright	-	-	-	-	+	-	+	CD56bright
17	492,009	bright	-	-	-	+	+	-	+	CD56bright
18	42,988	bright	+	-	+	-	+	-	+	CD56bright and NKG2C+
19	181,583	dim	+	+	-	+	-	-	-	CD56dim
20	273,266	dim	+	+	+	-	-	-	-	CD56dim and NKG2C+
21	23,394	dim	-	-	-	+	-	+	+	CD56dim
22	17,574	dim	-	-	-	+	+	+	+	CD56dim
23	222,765	dim	-	-	-	-	-	-	-	CD56dim
24	17,905	bright	-	-	+	-	+	-	+	CD56bright and NKG2C+

25	84,213	bright	-	-	+	+	+	-	+	CD56bright and NKG2C+
26	46,549	bright	-	-	-	+	-	-	low	CD56bright
27	73,682	bright	-	-	-	+	+	-	low	CD56bright
28	327,552	dim	+	-	+	-	-	-	-	CD56dim and NKG2C+
29	26,106	dim	-	-	+	-	-	-	+	CD56dim and NKG2C+
30	6,280	-	+	-	-	-	-	low	+	CD56-
31	44,625	dim	-	-	-	-	-	-	+	CD56dim
32	42,584	dim	-	-	-	-	-	+	low	CD56dim
33	66,657	dim	+	-	+	-	-	-	-	CD56dim and NKG2C+

Supplemental Table 4: Cell count, marker annotation and T-cell population for 37 clusters

		Marker													
Cluster	Cell count	TCRVd2	CD4	CD8	CD45RA	CCR7	CD95	CD27	CD28	CCR6	CXCR3	CD25	CD127	T cell population 1	T cell population 2
1	2,504,123	-	-	-	-/+	-/+	-/+	-/+	-/+	-/+	-/+	-	-/+	DN T cells	-
2	16,264,042	-	+	-	+	+	-	+	+	-	-	-	+	CD4 T cells	Tn CD4
3	1,109,850	-	+	-	+	+	-	+	+	-	-	+	-	CD4 T cells	Treg
4	7,785,873	-	-	+	+	+	-	+	+	-	-/+	-	+	CD8 T cells	Tn CD8
5	1,427,263	-	-	+	-	-	+	+	-/+	-	+	-	-/+	CD8 T cells	Ttm CD8
6	667,707	-	-	+	+	+	+	+	+	-	+	-	+	CD8 T cells	Tscm CD4
7	496,750	-	-	+	+	-	+	+	-	-	+	-/+	+	CD8 T cells	Temra CD8
8	111,744	-	+	-	+	+	+	+	+	-/+	+	-/+	-/+	CD4 T cells	Tscm CD4
9	245,538	-	+	-	+	+	-	+	+	-	+	-	+	CD4 T cells	Tn CD4
10	53.276	-	+	-	+	-	+	+	+	-/+	+	-/+	+	CD4 T cells	Temra CD4
11	21.127	-	+	-	+	+	+	+	+	-	-	-	-	CD4 T cells	Tscm CD4
12	1.931.523	-	-	+	-	-	+	+	+	+	-/+	-	+	CD8 T cells	Ttm CD8
13	1.554.760	-	+	-	-	+	+	+	+	-	-	-/+	+	CD4 T cells	Tcm CD4
14	238.059	-	+	-	-	-	+	+	+	-	-	-/+	+	CD4 T cells	Ttm CD4
15	685.024	-	-	+	+	-	-/+	+	-	-	-/+	-	-	CD8 T cells	Temra CD8
16	307,529	-	-	+	-	+	+	+	+	low/+	+	+	+	CD8 T cells	Tcm CD8
17	1,611,587	-	-	+	+	-	+	-	-	-	-/+	-	-	CD8 T cells	Temra CD8
18	490,180	-	-	+	-	-	+	+	-	-	-/+	-	-	CD8 T cells	Tem CD8
19	632,504	-	-	+	-	-	+	-	-	-	-/+	-	-	CD8 T cells	CD8+, CD45RA-, CCR7-, CD27-, CD28-
20	2,278,361	-	+	-	-	+	+	+	+	-	+	-/+	+	CD4 T cells	Tcm CD4
21	949,601	-	+	-	-	-	+	+	+	-	+	-/+	+	CD4 T cells	Ttm CD4
22	10,791	-	+	+	-	-/+	+	+	+	-	-	-/+	+	DP T cells	-
23	70,577	-	-	+	-	+	+	+	+	-	-	-/+	+	CD8 T cells	Tcm CD8

24	25,965	-	-	+	-	-	+	+	+	-	-	-/+	+	CD8 T cells	Ttm CD8
25	84,7667	-	+	-	-	-/+	+	+	+	-/+	-/+	+	-	CD4 T cells	Treg
26	307,928	-	+	-	-	-	+	-	-	-	-/+	-	-	CD4 T cells	CD4+, CD45RA-, CCR7-, CD27-, CD28-
27	177,860	-	-	+	-	-	+	-	+	+	-/+	-	+	CD8 T cells	CD8+, CD45RA-, CCR7-, CD27-, CD28+
28	1,265,483	-	+	-	-	-	+	-	+	-	-/+	-/+	+	CD4 T cells	Tem CD4
29	1,155,959	-	+	-	-	+	+	+	+	+	+	+	+	CD4 T cells	Tcm CD4
30	522,811	-	+	-	-	-	+	+	+	+	+	+	+	CD4 T cells	Ttm CD4
31	1,176,983	-	+	-	-	+	+	+	+	+	-	+	+	CD4 T cells	Tcm CD4
32	169,533	-	+	-	-	-	+	+	+	+	-	+	+	CD4 T cells	Ttm CD4
33	406,018	-	+	-	-	-	+	-	+	+	+	+	+	CD4 T cells	Tem CD4
34	362,257	-	+	-	-	-	+	-	+	+	-	+	+	CD4 T cells	Tem CD4
35	333,288	+	-	+	-/+	-	-	+	-/+	-	+	low	+	γδ T cells	-
36	3,042,351	+	-	-	-/+	-	-	+	-/+	-	+	-	+	γδ T cells	-
37	1,196,647	+	-	-	-	-	+	+	+	low/+	+	low	+	γδ T cells	-

Supplemental Table 5: Cell count, marker annotation and B-cell population for 31 clusters

Cluster	Cell count	Marker												B cell population
		IgD	IgM	IgA	CD27	CD21	CD38	CD24	CD11c	CCR7	CCR6	CXCR5	CXCR3	
1	113,849	-	-	+	+	+	low	+	-	+	+	+	-	Switched memory B cells
2	41,077	-	-	+	+	+	low	low	-/+	-	+	+	-	Switched memory B cells
3	30,013	-	-/+	-	+	-	high	-	-	-	-	-	-	Plasmablasts
4	27,104	-	-	+	+	-	high	-	-	-	-	-	-	Plasmablasts
5	29,758	-	-	-	-	-	-	-	+	-	+	-	+	Switched memory B cells
6	22,410	-	-	-/+	low	-	high	-	-	-	-	-	-	Plasmablasts
7	3,089,971	+	+	-	-	+	low	low	-	+	+	+	-	Naïve B cells
8	19,772	+	-	-	-	-	-	-	-/+	-	+	-	+	IgD-only B cells
9	50,271	+	+	-	-	-	+	+	-	-	low	+	-	Transitional B cells
10	175,072	+	+	-	-	+	high	high	-	-	low	-/+	-	Transitional B cells
11	38,086	-	-	+	+	+	low	+	-	+	+	+	+	Switched memory B cells
12	69,857	-	-	-	+	+	-	low	-	-	+	+	-	Switched memory B cells
13	138,813	-	-	-	+	+	low	+	-	+	+	+	+	Switched memory B cells
14	52,186	+	-	-	-	+	-	-	-	-	-	+	-	IgD-only B cells
15	21,621	-	-	-	-	-	-	-	-	-	-	low	-	Switched memory B cells
16	28,476	-	-	-	+	-/+	low	low	-/+	-	+	+	+	Switched memory B cells
17	55,557	-	-	-	+	+	-	high	-	-	+	+	-	Switched memory B cells
18	206,392	-	-	-	low	+	low	+	-	+	+	+	-	Switched memory B cells
19	13,268	-	-/+	-	+	+	+	-	low	-	+	low	-	Switched memory B cells
20	586,535	+	+	-	-	+	+	+	-	+	+	+	-	Transitional B cells
21	18,601	-	+	-	+	+	-	high	-	-	+	+	+	IgM-only B cells
22	20,245	-	+	-	+	+	low	high	-	+	+	+	-	IgM-only B cells
23	81,751	+	+	-	+	+	-	high	-	-	+	+	-	MZ-like B cells
24	338,391	+	+	-	low	+	-	+	-	+	+	+	-	Naïve B cells

25	25,560	+	+	-	+	+	-	high	low	-	+	+	+	MZ-like B cells
26	37,847	+	+	-	low	+	-	+	-	+	+	+	+	Naïve B cells
27	96,888	+	+	-	-	+	low	low	-	+	+	+	+	Naïve B cells
28	5,427	+	-	-	+	+	-	-/+	-/+	-	+	+	-/+	IgD-only B cells
29	14,453	+	+	-	-	low	-	-	-/+	-	+	low	+	Naïve B cells
30	20,324	+	+	-	-	+	-	+	-	-	+	+	+	Naïve B cells
31	14,056	+	+	-	-	+	+	+	-	-	+	+	+	Transitional B cells

Supplemental Table 6: Frequency and annotation of different immune cell populations in CYP with and without LC.

Cell type	Annotation	**LC cohort** % of total PBMCs (median, IQR)	**Control cohort** % of total PBMCs (median, IQR)	**LC cohort** % of parent (median, IQR)	**Control cohort** % of parent (median, IQR)
Monocytes	(SSC^{-}A/FSC^{-}A) CD14$^{+/-}$, CD16$^{-/+}$	10.15 (7.61, 12.6)	11.51 (10.00, 16.74)	-	-
Classical monocytes	CD14^{+}, CD16^{-}	-	-	94.71 (93.30, 96.23)	95.05 (93.46, 96.70)
Intermediate monocytes	CD14^{+}, CD16^{+}	-	-	2.91 (1.95, 3.98)	3.02 (1.96, 4.45)
Non-classical monocytes	CD14^{-}, CD16^{+}	-	-	2.18 (1.40, 2.85)	1.50 (0,65, 2.26)
Dendritic Cells	CD3^{-}, CD19^{-}, HLADR^{+}, CD14$^{-/+}$, CD16^{-}		1.65 (1.45, 1.81)	-	-
cDCs	CD14$^{-/+}$, CD16^{-}, CD11c^{+}, CD123^{-}	-	-	38.62 (32.32, 46.16)	34.46 (25.68, 42.62)
pDCs	CD14^{-}, CD16^{-}, CD11c^{-}, CD123^{+}	-	-	34.17 (27.26, 42.80)	40.42 (35.87, 47.16)
CD11c^{-}, CD123^{-} DCs	CD14^{-}, CD16^{-}, CD11c^{-}, CD123^{-}	-	-	19.84 (15.11, 24.86)	16.43 (13.41, 22.26)
NK cells	CD3^{-}, CD19^{-}, HLADR^{-}, CD14^{-}	9.29 (7.16, 13.19)	9.76 (5.81, 12.68)	-	-
Early NK cells	CD56^{bright}	-	-	10.23 (6.97, 14.92)	12.52 (8.46, 15.00)
Mature NK cells	CD56^{dim}	-	-	84.52 (78.21, 88.24)	82.85 (78.95, 84.69)
Exhausted NK cells	CD56^{-}	-	-	4.09 (2.67, 5.85)	3.95 (2.33, 5.73)
NKG2C^{+} memory NK cells	NKG2C^{+}	-	-	3.35 (1.41, 10.95)	1.98 (1.07, 5.68)
CD4 T cells	CD14^{-}, CD3^{+}, TCRVd2^{-}, CD4^{+}, CD8^{-}	31.57 (26.73, 35.67)	25.74 (21.08, 34.33)	-	-
CD4 Tn	CD45RA^{+}, CCR7^{+}, CD95^{-}	-	-	57.07 (48.38, 64.21)	53.24 (42.01, 57.95)
CD4 Tscm	CD45RA^{+}, CCR7^{+}, CD95^{+}	-	-	0.30 (0.22,0.43)	0.31 (0.24,0.42)
CD4 Tcm	CD45RA^{-}, CCR7^{+}	-	-	20.80 (15.61, 25.48)	23.90 (15.61, 25.48)
CD4 Ttm	CD45RA^{-}, CCR7^{-}, CD27^{+}, CD28^{+}	-	-	5.69 (4.05, 7.94)	6.06 (4.43, 9.77)
CD4 Tem	CD45RA^{-}, CCR7^{-}, CD27^{-}, CD28^{+}	-	-	6.42 (4.05, 7.94)	6.06 (4.43, 9.77)
CD45RA^{-}, CCR7^{-}, CD27^{-}, CD28^{-}	CD45RA^{-}, CCR7^{-}, CD27^{-}, CD28^{-}	-	-	0.11 (0.03, 0.94)	0.11 (0.06, 1.17)
CD4 Temra	CD45RA^{+}, CCR7^{-}	-	-	0.06 (0.04, 0.11)	0.07 (0.04, 0.10)
CD4 Treg	CD25^{+}, CD127^{-}	-	-	6.77 (5.55, 8.04)	6.30 (5.22, 7.26)

CD8 T cells	CD14⁻, CD3⁺, TCRVd2⁻, CD4⁻, CD8⁺	17.35 (14.80, 19.36)	19.01 (16.43, 21.81)	-	-
CD8 Tn	CD45RA⁺, CCR7⁺, CD95⁻	-	-	48.35 (38.29, 60.20)	42.96 (31.70, 54.98)
CD8 Tscm	CD45RA⁺, CCR7⁺, CD95⁺	-	-	3.63 (2.56, 4.77)	4.75 (3.36, 5.62)
CD8 Tcm	CD45RA⁻, CCR7⁺	-	-	2.03 (1.12, 3.46)	1.22 (0.65, 1.59)
CD45RA⁻, CCR7⁻, CD27⁻, CD28⁺	CD45RA⁻, CCR7⁻, CD27⁻, CD28⁺	-	-	0.69 (0.44, 1.44)	0.61 (0.46, 1.88)
CD8 Ttm	CD45RA⁻, CCR7⁻, CD27⁺, CD28⁺	-	-	20.31 (15.06, 25.88)	19.01 (14.08, 26.81)
CD8 Tem	CD45RA⁻, CCR7⁻, CD27⁺, CD28⁻	-	-	2.59 (1.68, 4.16)	2.80 (1.69, 4.03)
CD45RA⁻, CCR7⁻, CD27⁻, CD28⁻	CD45RA⁻, CCR7⁻, CD27⁻, CD28⁻	-	-	2.58 (1.28, 5.44)	3.09 (0.90, 8.47)
CD8 Temra	CD45RA⁺, CCR7⁻	-	-	13.51 (6.22, 21.14)	14.51 (11.06, 28.55)
γδ T cells	CD14⁻, CD3⁺, TCRVd2⁺	3.78 (2.38, 5.87)	4.00 (2.28, 6.91)	-	-
DN T cells	CD14⁻, CD3⁺, TCRVd2⁻, CD4⁻, CD8⁻	2.46 (1.98, 3.15)	2.78 (2.41, 3.84)	-	-
DP T cells	CD14⁻, CD3⁺, TCRVd2⁻, CD4⁺, CD8⁺	0.007 (0.004, 0.014)	0.006 (0.004, 0.007)	-	-
B cells	CD3⁻, CD19⁺, CD14⁻	5.91 (4.73, 7.52)	8.13 (4.67, 9.52)	-	-
Transitional B cell	IgD⁺, IgM⁺, CD27⁻, CD38⁺/ʰⁱ, CD24⁺/ʰⁱ	-	-	12.85 (9.42, 18.04)	16.46 (12.09, 21.62)
Naïve B cell	IgD⁺, IgM⁺, CD27⁻, CD38⁻/ˡᵒ	-	-	65.77 (58.83, 70.72)	69.97 (61.01, 73,85)
MZ-like B cell	IgD⁺, IgM⁺, CD27⁺, CD21⁺	-	-	1.32 (0.76, 1.98)	0.63 (0.48, 1.49)
IgM-only B cell	IgM⁺, IgD⁻, CD27⁺	-	-	0.64 (0.41, 1.04)	0.55 (0.39, 0.66)
IgD-only B cell	IgD⁺, IgM⁻	-	-	1.56 (1.15, 2.65)	1.59 (0.91, 2.31)
Switched memory B cells	IgD⁻, IgM⁻, CD38⁻/⁺	-	-	13.19 (9.90, 18.32)	10.77 (9.15, 12.94)
Plasmablasts	IgD⁻, IgM⁻, CD38ʰⁱ	-	-	1.05 (0.66, 1.80)	0.91 (0.70, 1.80)

Supplemental Table 7: Coefficients and p-values of linear regression models

	Coefficients				LRT p values			
	**Age**	**Sex=Male**	**Exposure= Two exposures**	**Group=PASC**	**Age**	**Sex**	**Exposure**	**Group**
**S2 IgG**	0.024	-0.091	0.711	0.061	0.235	0.418	6.12E-07	0.669
**RBD IgG**	0.014	0.269	1.109	-0.743	0.562	0.066	4.41E-10	8.99E-05
**S2 IgA**	0.039	-0.001	0.318	-0.061	0.038	0.925	0.011	0.652
**RBD IgA**	0.013	0.013	0.728	-0.310	0.549	0.915	2.01E-06	0.046
**Neutralization**	0.039	0.204	0.568	-0.330	0.192	0.112	1.47E-04	0.048

Supplemental Table 8: SARS-CoV-2 antigen and RNA persistence in CYP with and without long COVID

ID	Cohort	Spike (fg/mL)	Nucelocapsid (fg/mL)	3'UTR SARS-CoV-2 (copies/mL)	N SARS-CoV-2 (copies/mL)	Ng SARS-CoV-2 (copies/mL)	Nsg SARS-CoV-2 (copies/mL)
1	Long COVID	-	-	-	-	-	-
2	Long COVID	-	842	-	-	-	-
3	Long COVID	-	-	-	-	-	-
4	Long COVID	-	-	-	-	-	-
6	Long COVID	-	-	-	-	-	-
7	Long COVID	-	-	-	-	-	-
8	Long COVID	-	-	-	-	-	-
9	Long COVID	-	-	-	-	-	-
10	Long COVID	-	-	-	-	-	-
12	Long COVID	-	-	-	-	-	-
14	Long COVID	-	-	-	-	-	-
15	Long COVID	-	11,944	-	-	-	-
17	Long COVID	-	-	-	-	-	-
19	Long COVID	-	-	-	-	-	-
20	Long COVID	-	-	-	-	-	-
21	Long COVID	-	-	-	-	-	-
23	Long COVID	-	-	-	-	-	-
24	Long COVID	-	-	-	-	-	-
25	Long COVID	-	-	-	-	-	-
26	Long COVID	-	-	-	-	-	-
27	Long COVID	260	-	-	-	-	-
28	Long COVID	262	-	-	-	-	-
29	Long COVID	-	-	-	-	-	-
30	Long COVID	-	-	-	-	-	-
31	Long COVID	-	-	-	-	-	-

33	Long COVID	-	-	-	-	-	-
34	Long COVID	-	-	-	-	-	-
35	Long COVID	-	-	-	-	-	-
36	Long COVID	-	-	-	-	-	-
37	Long COVID	-	-	-	-	-	-
38	Long COVID	358	-	-	-	-	-
39	Long COVID	-	-	-	-	-	-
40	Long COVID	267	-	-	-	-	-
42	Long COVID	-	-	-	-	-	-
43	Long COVID	-	-	-	-	-	-
44	Long COVID	-	-	-	-	-	-
45	Long COVID	-	-	-	-	-	-
46	Long COVID	-	-	-	-	-	-
47	Long COVID	-	-	-	-	-	-
48	Long COVID	-	-	14	-	-	-
49	Long COVID	-	-	-	-	-	-
50	Long COVID	-	-	-	-	-	-
51	Long COVID	-	-	-	-	-	14
52	Long COVID	-	-	-	-	-	-
53	Long COVID	-	-	-	-	-	-
55	Long COVID	-	527	-	-	-	-
56	Long COVID	-	-	-	-	-	-
57	Long COVID	-	-	-	-	-	-
58	Long COVID	-	-	-	-	-	-
59	Long COVID	-	-	-	-	-	-
60	Long COVID	-	-	-	-	-	-
61	Long COVID	-	-	-	-	-	-
62	Long COVID	-	529	-	-	-	-

63	Long COVID	-	-	-	-	-	-
65	Long COVID	-	-	-	-	-	-
67	Long COVID	-	-	-	-	-	-
68	Long COVID	250	9,324	-	-	-	-
69	Long COVID	-	-	-	-	-	-
70	Long COVID	-	-	-	-	-	-
71	Long COVID	-	-	-	-	-	-
73	Long COVID	-	-	-	-	-	-
74	Long COVID	-	8,555	-	-	-	-
76	Long COVID	-	-	-	-	-	-
77	Long COVID	-	-	-	13	-	-
78	Long COVID	-	-	-	-	-	-
79	Long COVID	-	-	-	-	-	-
80	Long COVID	-	-	-	-	-	-
81	Long COVID	-	-	-	-	-	-
82	Long COVID	-	-	-	-	-	-
83	Long COVID	-	-	-	-	-	-
84	Long COVID	-	-	-	-	-	-
85	Long COVID	-	-	-	-	-	-
86	Long COVID	-	869	-	-	-	-
87	Long COVID	-	-	-	-	-	-
88	Long COVID	-	-	-	-	-	-
89	Long COVID	-	-	-	-	-	-
90	Long COVID	-	-	-	-	-	-
91	Long COVID	-	-	-	-	-	-
92	Long COVID	-	-	-	-	-	-
93	Long COVID	-	-	-	-	-	-
94	Long COVID	-	-	-	-	-	-

96	Long COVID	-	659	-	-	-	-
98	Long COVID	-	-	-	-	-	-
99	Long COVID	-	-	-	-	-	-
100	Long COVID	-	-	-	-	-	-
101	Long COVID	-	-	-	-	-	-
102	Long COVID	-	-	-	-	-	-
103	Long COVID	-	671	-	-	-	-
104	Long COVID	-	-	-	16	-	-
105	Long COVID	-	-	-	-	-	-
106	Long COVID	-	-	-	-	-	-
107	Long COVID	267	-	-	-	-	13
108	Long COVID	-	-	-	-	-	-
109	Long COVID	-	-	-	-	-	-
110	Long COVID	-	-	-	-	-	-
111	Long COVID	-	-	-	-	-	-
112	Long COVID	-	-	-	-	-	-
114	Long COVID	-	-	-	-	-	-
115	Long COVID	-	-	-	-	-	-
C1	Controls	-	-	-	-	-	-
C2	Controls	-	-	-	-	-	-
C3	Controls	-	-	-	-	-	-
C4	Controls	-	-	-	-	-	-
C5	Controls	-	-	-	-	-	-
C7	Controls	-	-	-	-	-	25
C8	Controls	-	-	-	-	-	-
C9	Controls	-	-	-	-	-	-
C10	Controls	-	-	-	-	-	-
C11	Controls	-	-	-	-	-	-

C13	Controls	-	-	-	-	-	-
C14	Controls	-	-	-	-	-	-
C16	Controls	-	-	-	-	-	-
C17	Controls	-	-	-	-	-	28
C18	Controls	-	-	-	-	-	-
C19	Controls	-	-	-	-	-	-
C20	Controls	-	-	-	-	-	-
C21	Controls	-	-	-	-	-	-

Supplemental Table 9: Spearman correlations of time since last antigen exposure

Group	Variable	Rho	P.value
Long COVID	CCR6.MFI.CD56bright.NK.cells	0.267	0.008
Long COVID	CD8.MFI.CD56-.NK.cells	-0.256	0.011
Long COVID	CXCR5.MFI.cDCs	-0.139	0.171
Controls	CCR6.MFI.Intermediate.monocytes	-0.368	0.178
Controls	CXCR5.MFI.Tem.CD4	-0.396	0.182
Controls	CD8.MFI.CD56..NK.cells	-0.243	0.382
Long COVID	CCR6.MFI.Classical.monocytes	-0.082	0.420
Long COVID	CCR6.MFI.Intermediate.monocytes	-0.082	0.421
Long COVID	CCR6.MFI.Monocytes	-0.076	0.458
Long COVID	CD1c.MFI.IgM.only.B.cells	-0.076	0.467
Long COVID	CD1c.MFI.MZ.like.B.cells	-0.070	0.503
Long COVID	CXCR5.MFI.Tem.CD4	0.028	0.788
Controls	CXCR5.MFI.cDCs	-0.061	0.832
Controls	CD1c.MFI.MZ.like.B.cells	-0.036	0.903
Controls	CCR6.MFI.CD56bright.NK.cells	-0.036	0.903
Controls	CD1c.MFI.IgM.only.B.cells	-0.021	0.944
Controls	CCR6.MFI.Classical.monocytes	-0.018	0.954
Controls	CCR6.MFI.Monocytes	-0.018	0.954

Supplemental Table 10: Mann-Whitney Test comparing the significant results between infected and uninfected (vaccinated) controls

Variable	p.value	p.adj
Cluster.19.B.cells	0.013	0.451
Cluster.15.B.cells	0.028	0.451
CCR6.CD56bright.NK.cells	0.040	0.451
CD4.Tcm	0.112	0.587
CD8.tcm	0.190	0.886
Cluster.6.NK.cells	0.254	1.000
CD4.T.cells	0.364	1.000
CD8.Tscm	0.364	1.000
IgD.only.B.cells	0.371	1.000
Non.classical.monocytes	0.371	1.000
Switched.memory.B.cells	0.440	1.000
CD8.CD56..NK.cells	0.440	1.000
cDCs	0.513	1.000
Cluster.3.T.cells	0.518	1.000
Cluster.25.T.cells	0.518	1.000
pDCs	0.594	1.000
Cluster.23.B.cells	0.679	1.000
Cluster.25.B.cells	0.679	1.000
Cluster.25.NK.cells	0.679	1.000
CD1c.MZ.like	0.679	1.000
Cluster.16.T.cells	0.699	1.000
Cluster.23.T.cells	0.699	1.000
Cluster.17.B.cells	0.768	1.000
Cluster.15.NK.cells	0.768	1.000
CXCR5.cDCs	0.768	1.000
Treg	0.797	1.000
Cluster.7.M.cells	0.859	1.000
Cluster.11.M.cells	0.859	1.000
Cluster.26.NK.cells	0.859	1.000
CD1c.IgM.only	0.859	1.000
CXCR5.Tem.CD4	0.898	1.000
Monocytes	0.953	1.000
DCs	0.953	1.000
CCR6.Intermediate.monocytes	0.953	1.000
CCR6.Classical.monocytes	1.000	1.000
CCR6.Total.monocytes	1.000	1.000

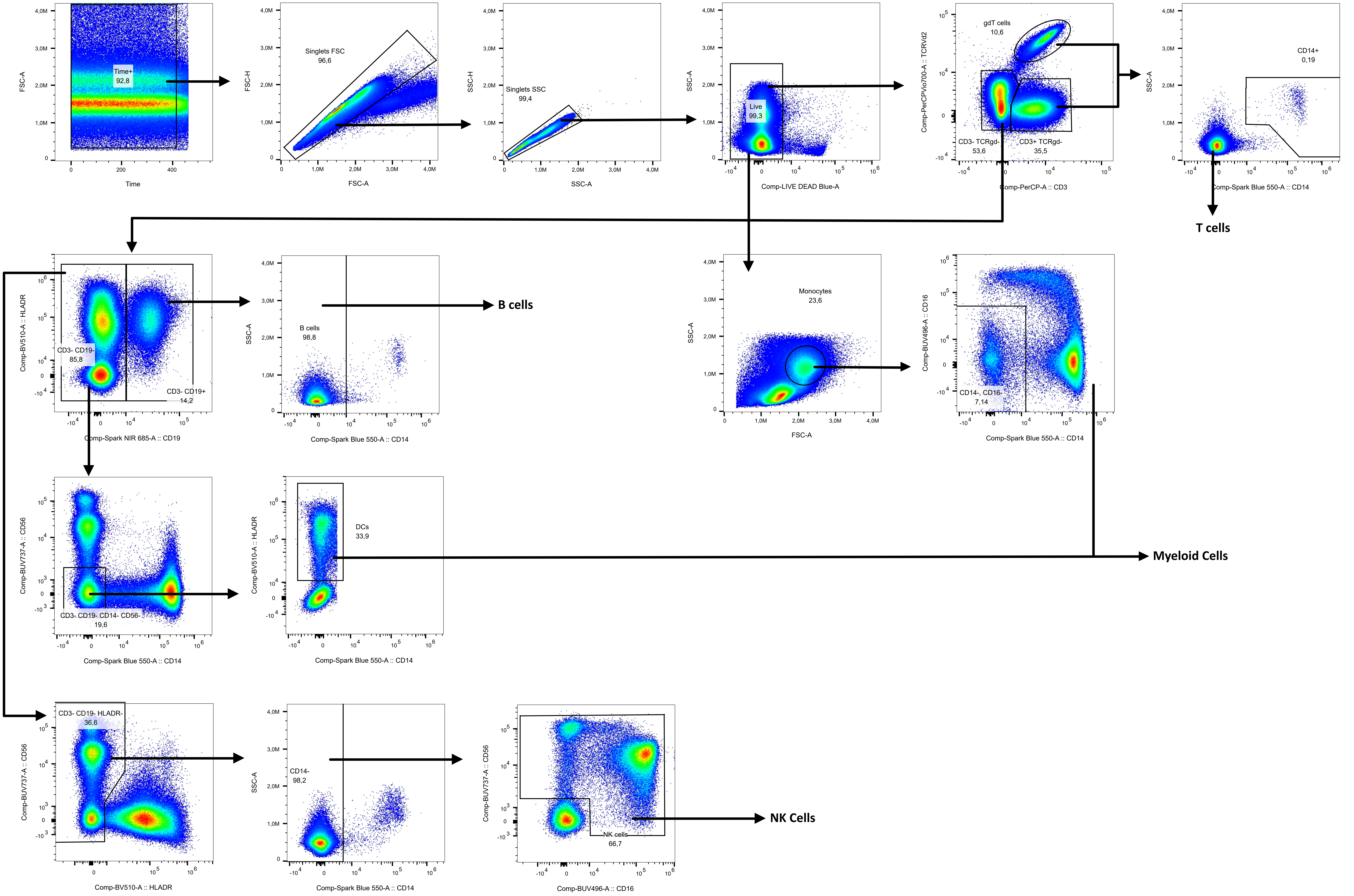

Supplemental Figure 1: Manual gating strategy

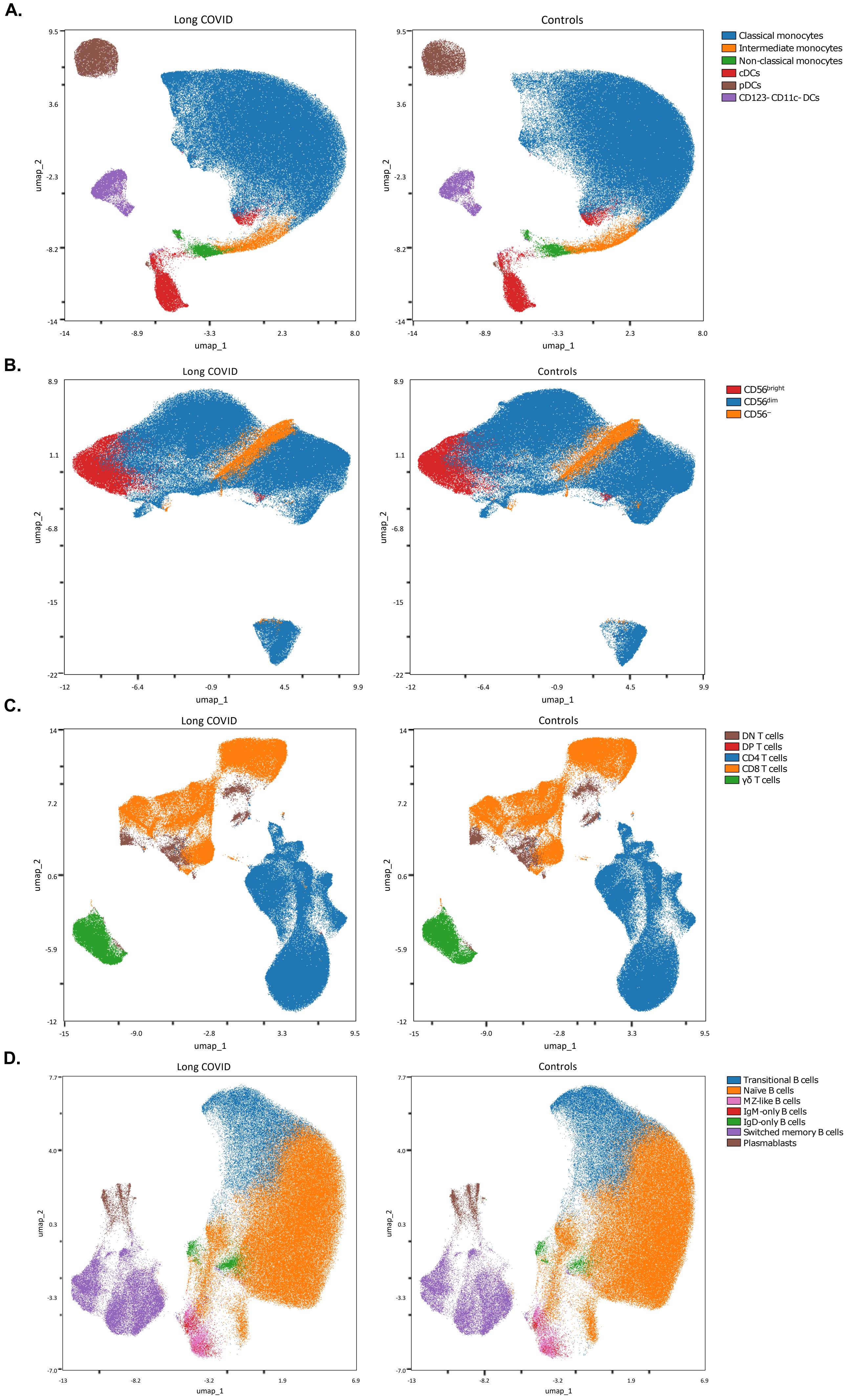

Supplemental Figure 2: UMAP representing all cell populations analyzed across the different major immune cell types. (A) Uniform manifold approximation and projection for dimension reduction (UMAP) representing the 6 major myeloid cell populations in which the 17 clusters analyzed using Flow-SOM were grouped depending on specific marker expression. **(B)** UMAP representing the 4 major NK-cell populations into which the 33 clusters analyzed using Flow-SOM were grouped depending on specific marker expression. **(C)** UMAP representing the 5 major T-cell populations into which the 37 clusters analyzed using Flow-SOM were grouped depending on specific marker expression. **(D)** UMAP representing the 6 major B-cell populations in which the 31 clusters analyzed using Flow-SOM were grouped depending on specific marker expression.

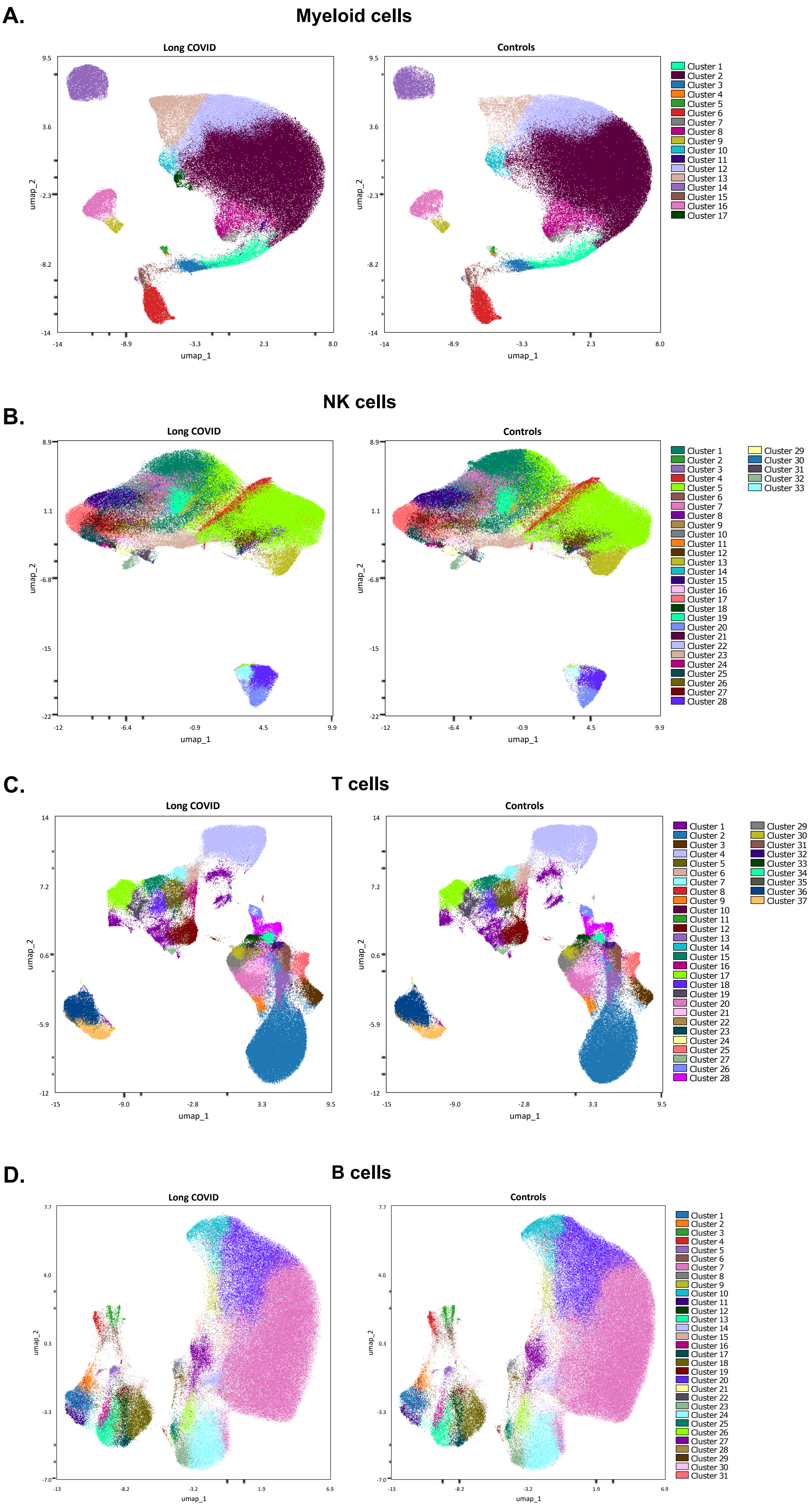

Supplemental Figure 3: UMAP representing all clusters analyzed across the different cell populations. (A) Uniform manifold approximation and projection for dimension reduction (UMAP) representing the 17 clusters analyzed in the myeloid cell population using Flow-SOM and grouped depending on specific marker expression. **(B)** UMAP representing the 33 clusters analyzed in the NK-cell population using Flow-SOM and grouped depending on specific marker expression. **(C)** UMAP representing the 37 clusters analyzed in the T-cell population using Flow-SOM and grouped depending on specific marker expression. **(D)** UMAP representing the 31 clusters analyzed in the B-cell population using Flow-SOM and grouped depending on specific marker expression.

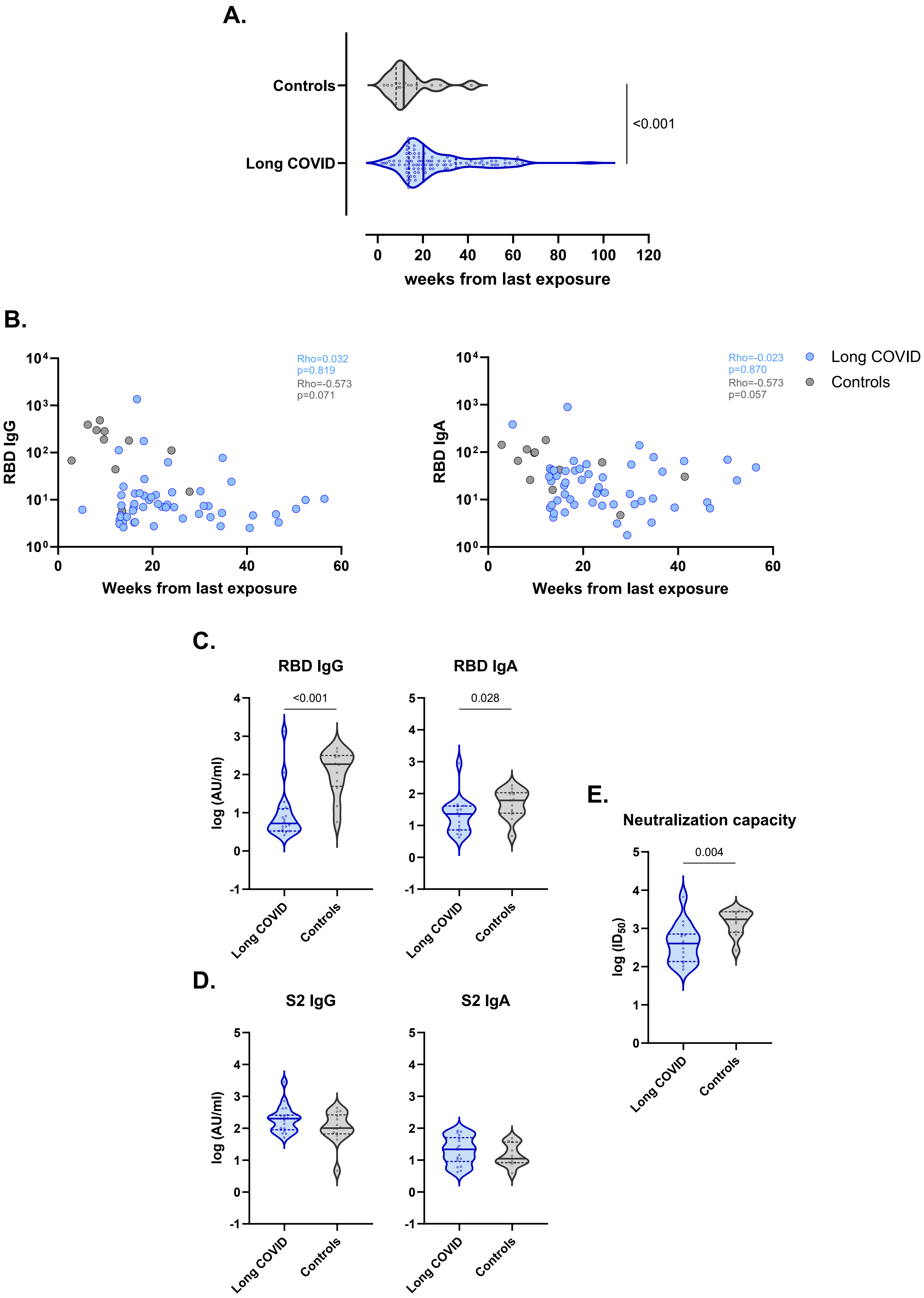

Supplemental Figure 4: Effect of time on antibody levels among CYP with and without LC. (A) Time to sample collection from the last exposure (weeks) in the LC and control cohort (median weeks [IQR]; LC: 20.3 [13.9-34.7]; controls: 11.6 [8.1-17.3]) ($p<0.001$). **(B)** Correlations of anti-RBD IgG (left) and anti-RBD IgA (right) in the LC and control cohorts (all p=n.s.) restricted to one-exposure samples. **(C)** Specific anti-RBD IgG (left) ($p < 0.001$) and IgA (right) ($p = 0.028$) antibody levels of responders. **(D)** Specific anti-S2 IgG (left) (p=n.s.) and IgA (right) (p=n.s.) antibody levels of responders. **(E)** Specific SARS-CoV-2 neutralizing capacity of responders in half-maximal inhibitory dilution (ID_{50}) among the LC and control cohort ($p = 0.004$). Analyses of antibody and neutralization levels were restricted to one-exposure samples with no differences in time from the last exposure (median weeks [IQR]; LC: 13.9 [13.4-16.1]; controls: 11.0 [8.3-21.8]). Each dot represents an individual, and median and IQR values are indicated. p-values according to the Mann-Whitney test. Significant p-values < 0.05.

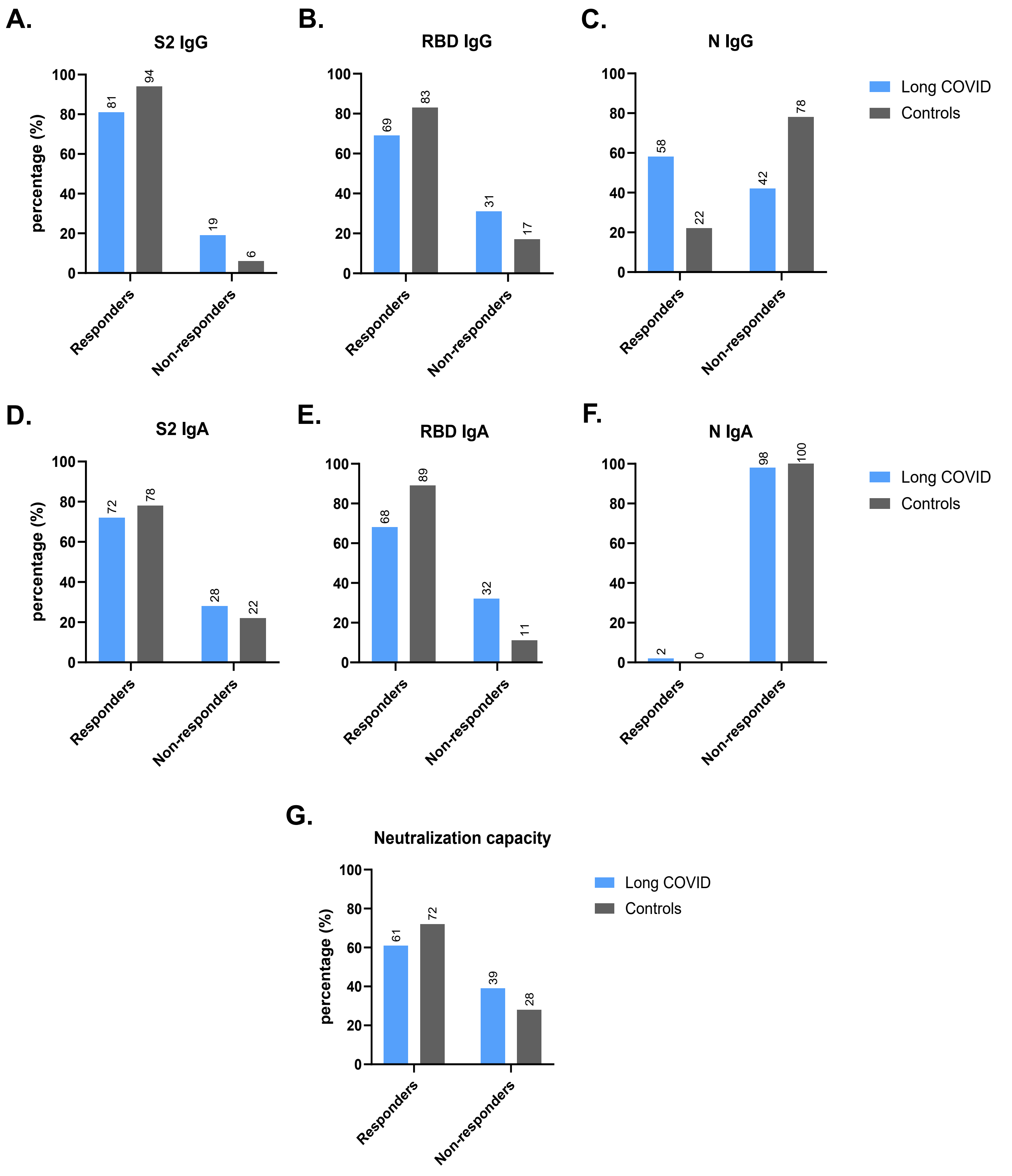

Supplemental Figure 5: Prevalence of responders and non-responders among CYP with and without LC. (A) Prevalence of anti-S2 IgG–specific responders and non-responders (%) among the LC and control cohorts. **(B)** Prevalence of anti-RBD IgG–specific responders and non-responders (%) among the LC and control cohorts. **(C)** Prevalence of Specific anti-N IgG responders and non-responders (%) among the LC and control cohorts. **(D)** Prevalence of Specific anti-S2 IgA responders and non-responders (%) among the LC and control cohorts. **(E)** Prevalence of specific anti-RBD IgA responders and non-responders (%) among the LC and control cohorts. **(F)** Prevalence of Specific anti-N IgA responders and non-responders (%) among the LC and control cohorts. **(G)** Prevalence of SARS-CoV-2–specific neutralizing capacity responders and non-responders (%) among the LC and control cohorts. LC n=99; controls n=18 in all analyses.

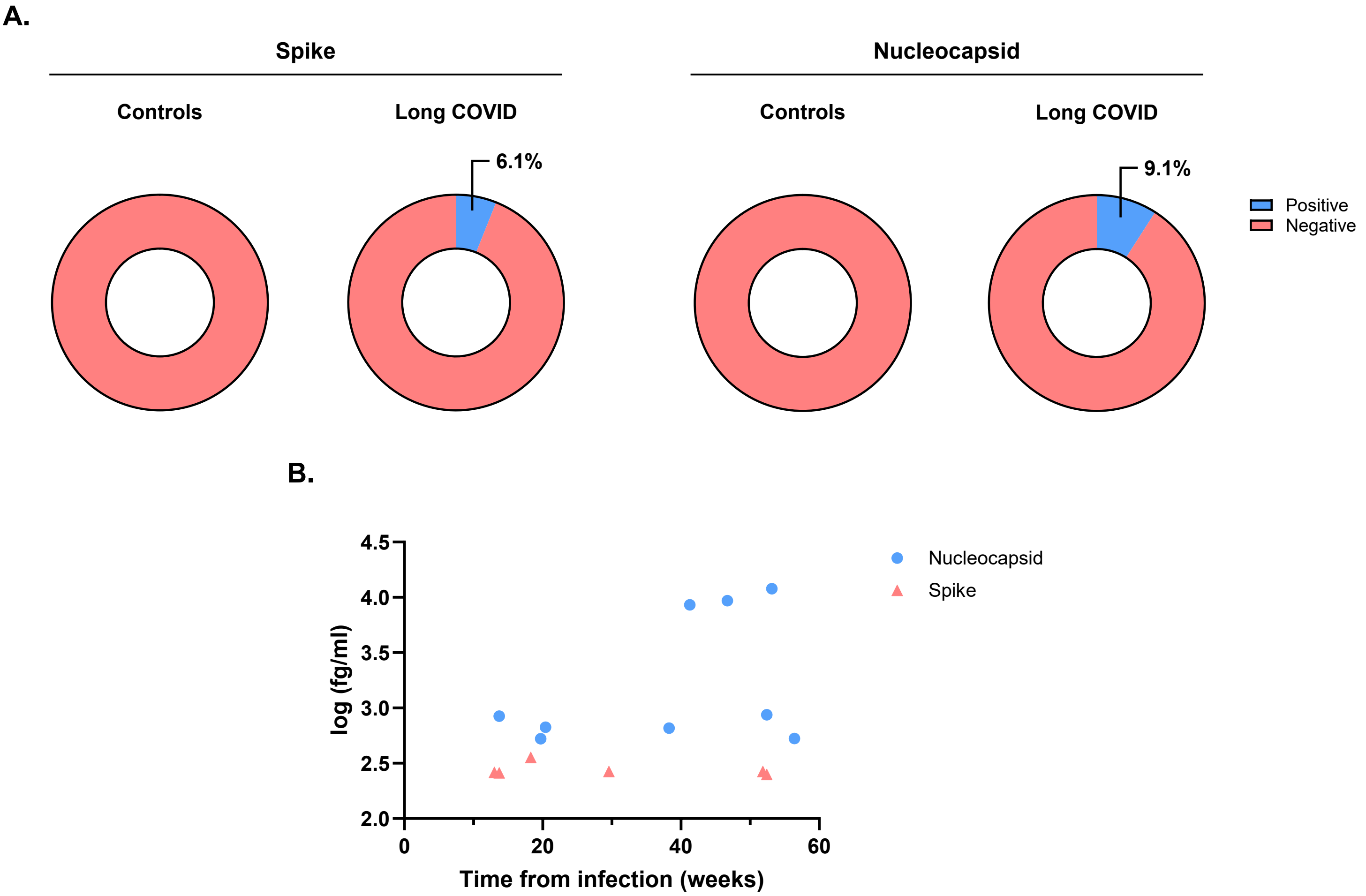

Supplemental Figure 6: SARS-CoV-2 antigen persistence in CYP with and without long COVID. (A) Percentage of antigen positivity of individuals with and without long COVID (right Spike, left Nucleocapsid). **(B)** Concentration of Spike (triangle, red) and Nucleocapsid (circle, blue) of positive samples in log(fg/ml) versus time from infection in weeks.

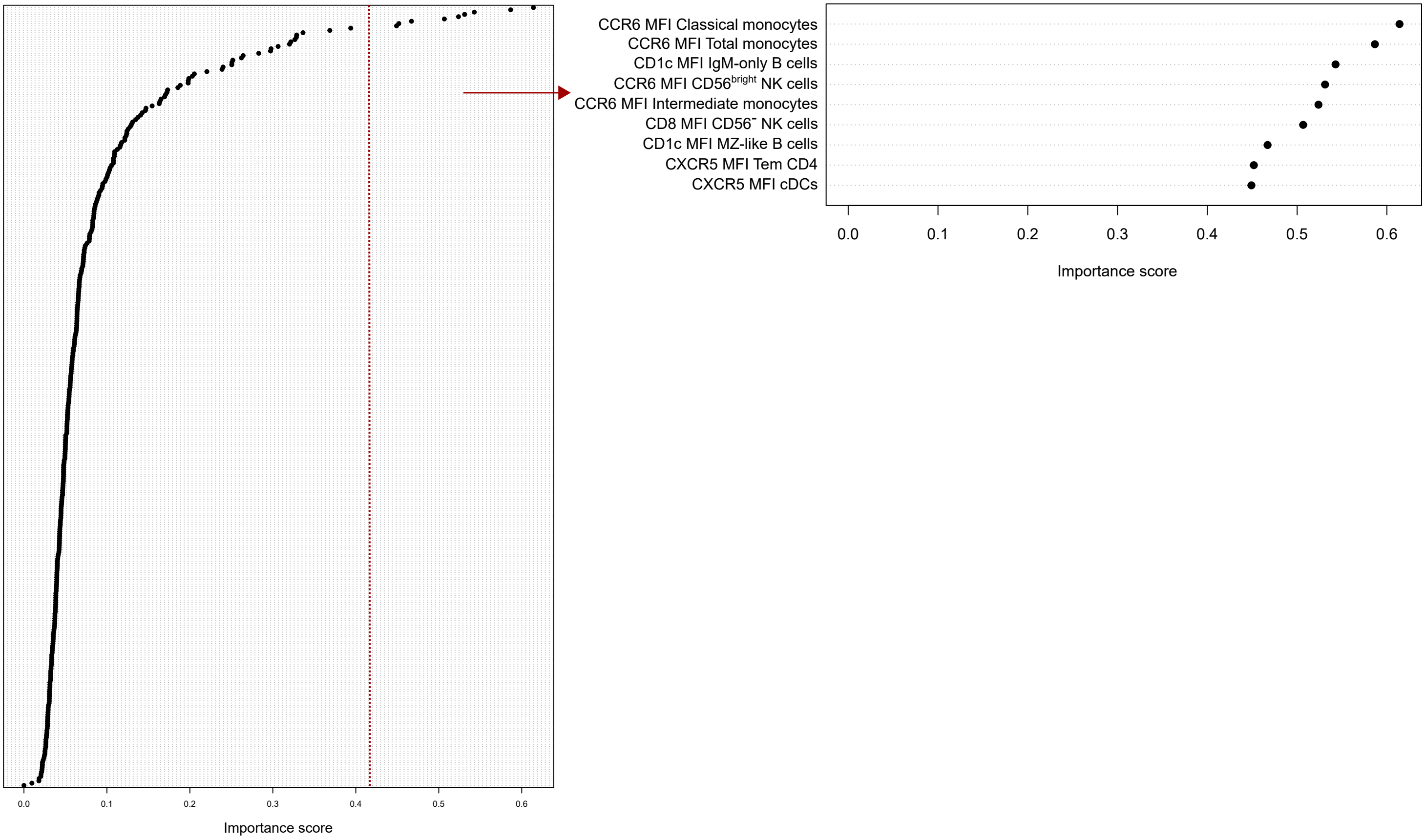

Supplemental Figure 7: Factor importance in random forest analysis. Relative variable importance of the factors included in the random forest analysis.

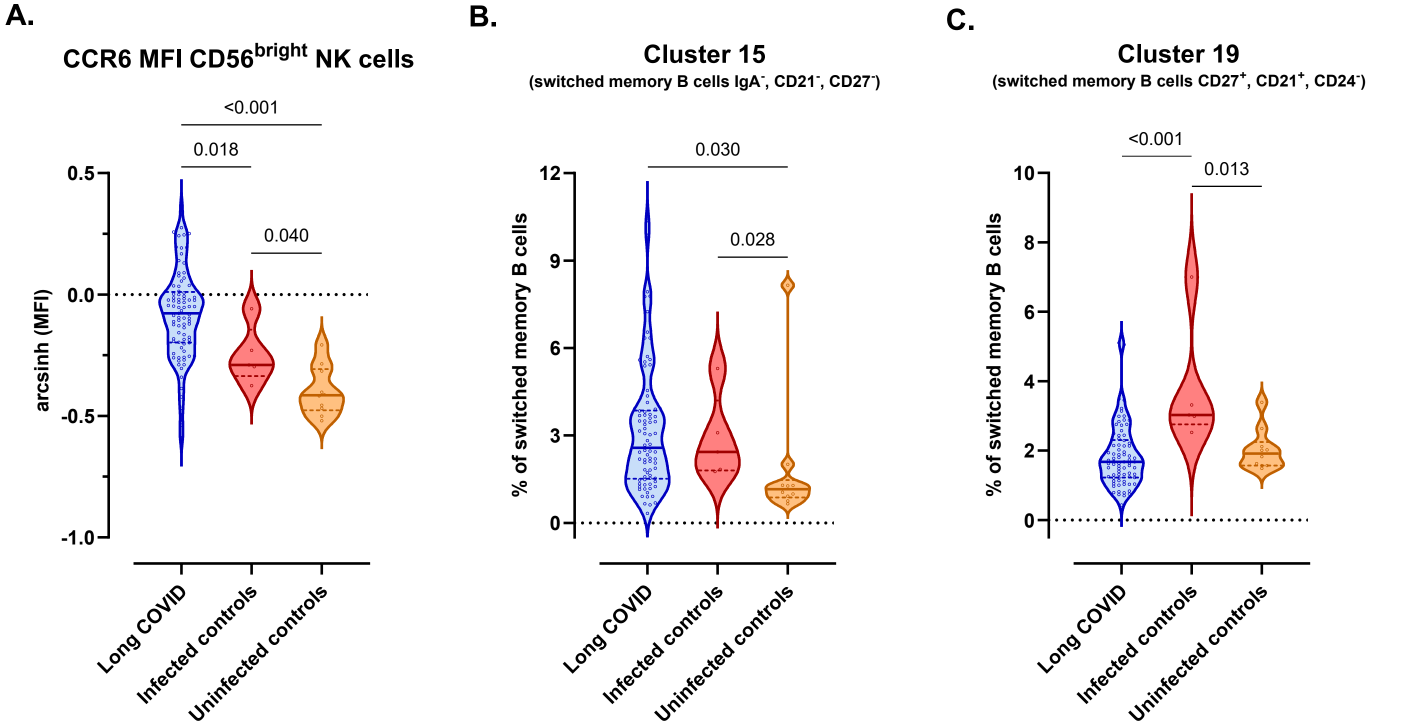

Supplemental Figure 8: Significant differences between infected and uninfected (vaccinated) controls. (A) Mean fluorescence intensity (MFI) of CCR6 in CD56bright NK cells. **(B)** Frequency of Cluster 15 with respect to total switched memory B cells. **(C)** Frequency of Cluster 19 with respect to total switched memory B cells. Mann-Whitney test, p-values < 0.05 were considered statistically significant.
